# Expressive voting versus information avoidance: experimental evidence in the context of climate change mitigation

**DOI:** 10.1007/s11127-022-01016-x

**Published:** 2022-12-31

**Authors:** Katharina Momsen, Markus Ohndorf

**Affiliations:** Department of Public Finance, University of Innsbruck, Innsbruck, Austria

**Keywords:** Expressive voting, Information avoidance, Experiment, Moral wiggle room, Climate change, C90, D12, D64, D72, D89, Q50

## Abstract

We theoretically and experimentally investigate the effect of self-serving information avoidance on moral bias in democratic and individual decisions in the context of climate change mitigation. Subjects choose between two allocations that differ in payoffs and contributions to climate change mitigation. We vary the observability of the environmental contribution, as well as the decision context associated with different levels of pivotality. If the contribution is directly observable, we find evidence for lower pivotality leading to higher levels of “green” decisions, as predicted by the low-cost theory of voting. This effect disappears if subjects can avoid information on the contribution. Instead, we find evidence for the exploitation of moral wiggle room via information avoidance in larger democracies as well as in the consumption context. Our results indicate that information avoidance substitutes expressive voting as an instrument to manage cognitive dissonance on the part of the voter. Hence, moral biases in elections might be less likely than previously thought.

**Supplementary information:**

The online version of this article (10.1007/s11127-022-01016-x) contains supplementary material, which is available to authorized users.

## Introduction

Decisions taken via democratic vote are often considered to yield more moral outcomes than individual decisions, in particular when it comes to choices made in a market context. This dichotomy between “electoral choice” and “market choice” (Brennan & Lomasky, 1993) is based on a compelling argument: According to the “low-cost theory of voting” (Tyran et al., 2019), instrumental considerations decrease with pivotality, i.e., the number of voters involved, while the utility from expressing moral principles remains unaffected by the size of the constituency. Thus, with decreasing probability of being pivotal, the ultimate decision is more likely to favor the more moral option (Brennan & Buchanan, 1984; Fiorina, 1976; Tullock, 1971). In market decisions, in contrast, the potential conflicts between instrumental and expressive motives seem to be often resolved in favor of the former, leading to more self-serving, less moral behavior. Recent experimental evidence seems to support this conjecture (Bartling et al., 2014; Falk & Szech, 2013; Falk et al., 2020).

From the outset, this dichotomy has been explained by the notion of decision-makers having to “reduce internal dissonance” (Tullock 1971, p. 387) between their moral preferences and payoff-oriented self-interest. This goes back to the theory of cognitive dissonance, first derived in Festinger (1957), which establishes different individual strategies to resolve such internal conflicts. For example, in situations where moral views and self-interest are in conflict, the individual can resolve this conflict by deciding in favor of either one option or the other. In the context of the low-cost theory of voting, the small probability of being pivotal alters this trade-off in favor of expressing moral principles, leading to more moral decisions.

Another strategy to reduce cognitive dissonance is to not use all available information on the consequences of a decision, even if information is accessible without cost. As was first pointed out by Festinger (1957), people tend to avoid information that might be incongruent with their established attitudes while disproportionately seeking news that is congruent—a tendency that is referred to as selective exposure to information.^1^ More recently, economists have identified this behavior as one of the most effective strategies for motivated reasoning to reach favorable conclusions on the effect of own actions on others (Bénabou & Tirole, 2016; Gino et al., 2016; Golman et al., 2017). In the simplest case, individuals can choose to remain entirely ignorant of the nature and scope of external effects that their own decisions have on others and choose according to their narrow self-interests. Thus, by avoiding information, they can circumvent feeling morally obliged to decide in favor of options associated with a larger positive (or a smaller negative) effect on others. They hence exploit moral wiggle room created via their deliberate lack of information (Dana et al., 2007). While there is ample experimental evidence of the exploitation of moral wiggle room in individual decision-making (e.g., Dana et al., 2007; Grossman & van der Weele, 2017; Momsen & Ohndorf, 2020), its importance in the context of incentivized voting decisions has not yet been investigated. In this paper, we present the results of a lab experiment designed to analyze the interactions between expressive voting and information avoidance. Hence, the paper closest to ours is Mechtenberg et al. (2021), which uses a field experiment to investigate moral self-signaling in voting. They find that moral self-image concerns do not lead to biases in information collection, but in information processing and voting behavior. While a dual-self approach has been shown by Grossman and van der Weele, (2017) to be a valid alternative to the more traditional concept of cognitive dissonance when it comes to explaining information avoidance in a formal model, it remains unclear how to apply this model to voting decisions. Hence, in its current form, Mechtenberg et al. (2021) refrains from presenting a formal model that integrates the dual self into the underlying voting game. We believe that the cognitive dissonance approach is better suited to such an integration, as shown within our formal considerations.^2^

In our experiment, third-party consequences are implemented as contributions to carbon offsets of different sizes, such that decisions in the lab affect climate-change mitigation. The climate change context seems to be particularly prone to the dynamics of information avoidance via self-selection into echo chambers and networks with like-minded individuals.^3^ In the contemporary academic discourse, such behavior is often associated with biases in information-seeking within social media networks (Bakshy et al., 2015; Halberstam & Knight, 2016). However, the problem of information avoidance is not limited to phenomena of online news consumption. As Boxell et al. (2017) report, polarization of political views might be more pronounced in demographic groups which are least likely to use the internet and social media. In cases where this choice is made willingly and is not compensated by a balanced consumption of traditional media, this would also qualify as a specific form of information avoidance (Klapper, 1960).

In order to examine the behavioral fundamentals of information avoidance, we investigate individual decisions and democratic votes under full information and compare these to a situation where information is initially unobservable but revealable without payoff-relevant cost. We derive predictions from a stylized model of voting decisions where the expressive term is specified as avoided costs from potential cognitive dissonance, which is in turn derived from the formalizations presented in Rabin (1994), Konow (2000), and Spiekermann and Weiss (2016). Formulating expressive voting as a matter of cognitive dissonance not only does justice to the above-cited origins of this theory, but also allows us to compare the effect of differing pivotality under full information to the one under potential information avoidance.^4^ To derive our hypotheses, we integrate this cognitive dissonance model into a simple Bayesian voting game with symmetric strategies and without abstention, which is based on Feddersen and Pesendorfer (1997) and Tyson (2016).

We test our theoretical predictions in an experiment implementing four different decision contexts: individual choice (framed as a consumption decision), majority voting in small and large groups (democracies), and randomized dictatorship. The consumption setting is included to take the initial juxtaposition of market choice and electoral choice made in Brennan and Lomasky (1993) into account.^5^ In all other treatment conditions, decisions affect groups with varying probabilities of a subject’s decision to be pivotal.

Under full information, we observe the lowest share of selfish choices in conflict situations in the individual choice treatment (consumption). In fact, the number of self-serving decisions in this treatment is significantly lower than that with majority voting in small groups and with the randomized dictator condition. Hence, in our experiment, the market context clearly does not lead to a less moral outcome—a finding that apparently contradicts the prediction with respect to markets made in the early literature on expressive voting, in particular in Brennan and Lomasky (1993).^6^ Yet, we surmise that the comparatively low number of “green” choices in the individual choice treatment could be attributed to the effect of rivaling other-regarding preferences in the group settings, as decisions also affect payoffs of other group members.^7^

Interestingly, when comparing our voting treatments under full information, we find a strong effect of pivotality, which is consistent with the low-cost theory of voting. The number of self-serving choices for the full information condition is significantly lower when voting in a larger group than for the treatments with a higher probability of being pivotal. This is in line with identifying a moral bias in larger elections presented in Shayo and Harel (2012), Fischer (1996), Feddersen et al. (2009), and Bischoff and Egbert (2013) and contrasts the studies that do not find an effect of expressive voting (Kamenica & Brad, 2012; Tyran, 2004; Tyran & Sausgruber, 2006).^8^ Framing the decision not as a voting situation, but as a (structurally equivalent) randomized dictatorship does not affect the direction of the effect of pivotality. Furthermore, this effect is stronger for smaller payoff differences, as predicted within our theoretical considerations.^9^

Yet, most importantly, in our treatments with hidden but revealable information, all differences in selfish choices disappear, which suggests that the effect of pivotality is dominated by information avoidance. Indeed, when analyzing the potential occurrence of self-serving information avoidance, we find that this phenomenon arises predominantly in those treatments that yield the largest number of “green” choices under full information, i.e., voting in larger groups and the individual choice condition. In these treatments, exploitation of moral wiggle room is also less pronounced if the difference in payoffs is large, which is in line with our theoretical predictions.^10^ Given these results, we conclude that avoiding information is the preferred strategy to deal with cognitive dissonance over all decision contexts investigated here, effectively dominating other strategies, like expressive voting. This result suggests that moral biases might be less likely in elections than previously thought.

The remainder of the paper is organized as follows: The following section describes our experimental design. We derive our behavioral predictions theoretically in Sect. 3. Our results are laid out in Sect. 4. The last section concludes. A translation of the experimental instructions as well as supplementary analyses are relegated to the Appendix.

## Experimental design

To investigate the effects of pivotality, information avoidance, and their interaction, we conducted a laboratory experiment where subjects made climate-relevant decisions. The experiment consisted of eight treatments, with each treatment capturing a different decision situation with respect to information and pivotality. In each treatment, subjects made 24 consecutive binary decisions between two allocations that differed in their payoffs and in their contributions to climate change mitigation. The latter was implemented via contributions to reduce global greenhouse gas emissions through the acquisition of high-quality carbon offsets on the voluntary offset market. Hence, decisions made in the experiment had an impact beyond the laboratory.^11^

Subjects could choose between two options, A and B. The payoffs of the options were drawn from a range of 10 to 90 European Currency Units (ECUs), and within a round, the payoff difference between the two options could be 5, 10, 15, or 20 ECUs. For option A, the contribution to the carbon offset was always equal to 15 ECUs. The contribution associated with option B could be either 0 or 30 ECUs, with each outcome being equally likely. For each round, the associated contribution was independent of the one realized in the previous round, such that subjects should consider each decision separately. The order in which subjects faced the decision situations was randomized, i.e., decision situations were randomly drawn from a pool of possible decision situations without replacement. We implemented two different types of decision situations: In situations with aligned interests, the option with the higher payoff for the subject(s) generated the larger contribution to the carbon offset, whereas in situations with conflicting interests, the option with the lower payoff was associated with a larger contribution. Both types of situations were equally likely to occur, and the order was randomized.

### Treatments

The experiment consisted of eight between-subjects treatments implemented in a 2 $\times$ 4 design, varying both the observability of option B’s externality and the decision context.

#### Information conditions

In the treatments with “full” information, the contributions to offsets of options A and B were immediately observable. Since the payoffs of both options were also depicted, subjects were immediately aware of the type of situation, i.e., if they were to make a decision with conflicting or aligned interests. In the treatments with “hidden” information, the contribution to offsets associated with option B was initially unobservable but could be revealed by clicking a button. Clicking the button was associated with a nominal cost of 0.1 ECUs, which was, in fact, not payoff-relevant: With an exchange rate of 10 ECUs to 1 euro, the costs of revealing information were equal to 1 cent. Yet, final earnings in Euros were rounded up to the next 10 cents, of which the subjects were informed in the instructions. Since only two rounds were payoff-relevant, they could infer that the costs of clicking could not reduce their final payoff. However, even if they did not engage in these computations, they could immediately see that the cost of 0.1 ECUs was very low. These small, only nominal costs were included to capture the fact that information on externalities is often available, but it takes a negligible amount of effort to gather, which may be taken as an excuse to remain ignorant. Clicking the button was optional, i.e., subjects could make their allocation decision without knowing if they were in a situation with conflicting or aligned interests.

#### Decision contexts

In addition to varying the availability of information on the externalities, we altered the institutional setting in which decisions were made. First, in order to reflect the initial juxtaposition by Brennan and Lomasky (1993), we included an individual choice situation which was framed as a consumption decision. Hence, the individual choice condition was similar to the design used in Momsen and Ohndorf (2020, 2022) with subjects taking the role of buyers, while the supply side of the market was computerized.^12^ In each round, subjects had to decide which of the two virtual products to purchase. They were endowed with 100 ECUs, which they could spend on one of the two products. As described above, the product options differed in their prices and the associated contribution to offsets. The subjects’ payoff resulted from their endowment minus the price paid for the selected product.^13^ Both the price of the option and the resulting payoff were displayed on their screens. Subjects did not have the option not to purchase. In contrast to the other decision contexts, decision-making in this institutional setting was entirely individual.

In the “voting” settings, subjects were split into groups which consisted—depending on the treatment—of 3 or 11 group members. Subjects had to vote on the option they wanted to implement for all members of their group. The option receiving the majority of votes was implemented. If option A received the majority of votes, each group member contributed 15 ECUs to the carbon offset and earned the payoff associated with option A. After each round, new groups were randomly formed.^14^

As potential moral bias in the voting treatments might be sensitive to a “democracy frame,” we also included a “Randomized Dictator” setting, where subjects were also split into groups of three. In each group, a randomly determined “dictator” decided which option to implement for all group members. If, for example, the “dictator” decided in favor of option A, each group member earned the payoff associated with option A and contributed 15 ECUs to the carbon offset. Hence, in total, the group contributed 45 ECUs. The other two group members also made allocation decisions which remained hypothetical. The identity of the decision-maker responsible for the allocation decision of a group was determined randomly after all group members had made their allocation decisions. Thus, the probability of being pivotal for each subject was 1/3. In each round, new groups were formed and subjects remained anonymous throughout the experiment.

In the individual choice condition under full information, each participant knew after each round how much she had earned and how much she had contributed to the carbon offset. To ensure comparability between the different institutional settings, we also provided feedback to the participants in the Voting and in the Dictator treatments after each round on how much they earned and how much was contributed to the carbon offset on their behalf. Note that this feedback was also provided under hidden information to ensure that the only difference between the information conditions lies in the information available before subjects make their decisions. Hence, potential dynamics should be identical across treatments.^15^

### Experimental procedure

The sessions for the experiment were run in the Innsbruck EconLab in October and November 2019. The experiment was programmed in zTree (Fischbacher, 2007) and participants were invited using hroot (Bock et al., 2014). As indicated in Table 1, 48 subjects participated in each treatment, with the exception of the Voting11 treatment where only 44 subjects participated. We ran two sessions for each treatment. In total, 376 subjects—mainly undergraduate students from all fields—participated in eight between-subjects treatments, earning on average €8.78. The average amount invested in carbon offset projects for each subject was €2.60. Hence, a total amount of €978 was used to purchase high-quality carbon offsets.

At the beginning of each session, subjects received printed instructions, which were read out aloud to create common knowledge. Afterwards, a short quiz on the understanding of the instructions followed. As soon as all subjects had completed the quiz successfully, they made their first allocation decision without knowing how the experiment would continue. After the first round, they received new instructions informing them that 23 additional rounds would follow. This design feature allows us to treat the first round as a one-shot decision, which cannot be polluted by potential time trends. Both the first round and one randomly determined round of the following 23 rounds were payoff-relevant. The payoffs of these two rounds were added and converted into Euros using an exchange rate of 0.1. Sessions were concluded with an unincentivized questionnaire which elicited the subjects’ demographics as well as their political and environmental preferences. Subjects received their payoff privately and in cash at the end of a session which lasted approximately 35 minutes including payment.

**Table 1:** Participants per treatment

	Full information	Hidden information
Ind. choice	48	48
Dictator	48	48
Voting3	48	48
Voting11	44	44
Total	188	188

## Behavioral predictions

For both phenomena investigated in our experiment, expressive voting and self-serving information avoidance, explanations have been brought forward based on the concept of cognitive dissonance (Festinger, 1957). In our experimental decision situations, such cognitive dissonance can arise if a person experiences a conflict between the motive of maximizing their own monetary payoff and the ideal of providing high levels of environmental benefits. If the effects of their own actions are known, as in our full information treatments, the subject needs to resolve this conflict by either deciding not to maximize their monetary payoff or by not corresponding to their environmental ideals. Both strategies are associated with either monetary or psychological costs, such that the resolution of the cognitive dissonance is always associated with trade-offs. In a group decision with pivotality lower than 1, this trade-off is altered. If the probability of being decisive in a voting context is low enough, the expected impact of the own vote on the monetary outcome is small. In such cases, voting for the “greener” option to correspond to own ideals is associated with lower expected monetary costs. The cognitive dissonance is hence resolved by choosing the green option and the expressive motive of voting dominates considerations on instrumental utility. This reasoning is at the basis of the low-cost theory of voting, predicting that for larger constituencies—and hence lower probabilities of casting the pivotal vote—the number of “moral” votes will be larger than for smaller constituencies or individual decisions (Brennan & Lomasky, 1993; Feddersen et al., 2009; Shayo & Harel, 2012; Tyran, 2004).

If information on the effects of the own decision needs to be revealed by the individual, there exists an additional strategy to reduce cognitive dissonance, as they can choose to only reveal information that is congruent with their ideals. This phenomenon is referred to as selective exposure to information (Festinger, 1957). In its simplest form, a person would simply avoid revealing information on the moral implications of their decision to exploit moral wiggle room (Dana et al., 2007). If the consequences of own decisions for third parties or the environment are unknown, the size of the cognitive dissonance is reduced. In such a case, the individual’s self-image is only challenged by the possibility, not necessarily the fact, that maximizing payoffs corresponds to the less moral option. In our hidden information treatments, it is a priori unclear whether the alternative with the higher monetary payoff is associated with the environmental benefit that is larger (aligned interests) or lower (conflicting interests). Thus, avoiding information and choosing the self-serving option can be a viable alternative to revealing the information and then choosing the greener option even if it is more expensive to do so.

In order to structure our understanding of these different phenomena and to add some rigor to our predictions, it is useful to derive a formal representation of the decisions taken within the experimental treatments presented above. Historically, economic formalization of cognitive dissonance has focused on the strategy of adjusting beliefs and fairness considerations (Konow, 2000; Rabin, 1994; Spiekermann & Weiss, 2016). In the following, we extend this model to also reflect the effects of pivotality and information avoidance in voting decisions. Our main focus is on the context of climate-relevant decisions as implemented in our experimental treatments.

### Individual decision

Consider the binary choice at the basis of our experiment. Denote with $\omega _B\in \{\underline{\omega }_B,\overline{\omega }_B\}$ the realized amount of offset contribution for Option B and with $\omega _A$ the certain amount associated with Option A, with $\underline{\omega }_B<\omega _A<\overline{\omega }_B$. The largest achievable offset contribution *d* is hence defined by $d=\textrm{max}\{\omega _A,\omega _B\}$. An individual with green preferences would prefer the option associated with *d* if her costs *c* associated with each option were equal (i.e., $c_B=c_A$). However, both options differ in costs to the individual, with the self-serving option denoted *s* for which $c_s=\textrm{min} \{c_A,c_B\}$.

In the case of individual choices under full information, cognitive dissonance occurs if individuals with (sufficiently intensive) green preferences choose the self-serving product option *s*, while $\omega _s<\omega _{\lnot s}=d$, i.e., if interests are conflicting. We follow Rabin (1994) by defining the costs from this dissonance when choosing option *i* as a function $\Phi (d-\omega _i;\alpha )$, with $\Phi (0)=0$, $\Phi '>0$, $\Phi ''>0$, and parameter $\alpha$ representing the intensity of the green preference with $\frac{d \Phi }{d\alpha }>0$. Under complete information, and for an endowment *m*, the individual’s payoffs for both product options are

1 $$\begin{aligned} U_s&=m-c_s-\Phi (d-\omega _s), \end{aligned}$$

2 $$\begin{aligned} U_{\lnot s}&=m-c_{\lnot s}-\Phi (d-\omega _{\lnot s})= m-c_{\lnot s}. \end{aligned}$$

Hence, under complete information, the selfish option is strictly dominated iff

3 $$\begin{aligned} \Phi (d-\omega _s)>\Delta _c=c_{\lnot s}-c_s. \end{aligned}$$

Thus, for $\Delta _c$ small enough, the individual will choose the more expensive product to avoid the cognitive dissonance associated with a self-image as an environmentally conscious individual. However, for larger levels of $\Delta _c$, the individual will accept the dissonance with respect to their self-image in favor of their narrow self-interest.

Let us now introduce the possibility of information avoidance as an additional strategy to reduce cognitive dissonance, as in our individual choice treatment with hidden information. For this, we assume the true value of $\omega _B$ to be initially unobservable, which implies that it is a priori unclear if interests are aligned or conflicting. Denote with $\mu$ the ex ante probability of interests being aligned, i.e., $\mu =P(\omega _s=d)$. Furthermore, we use index $k\in \{0,1\}$ to denote the state of the individual’s level of information, with $k=1$ representing a situation where the information is revealed, while $k=0$ represents non-revelation. Hence, for an uninformed individual $(k=0)$ for which (3) holds, the expected costs of cognitive dissonance $\Phi _0$ when choosing option *s* are determined by the individual’s (subjective) beliefs on the probability of aligned interests as follows:

$$\begin{aligned} \Phi _0=\Phi \left( \hat{E}(d)-\hat{E}(\hat{\omega }_s);\alpha \right) \end{aligned}$$

Notice that even without subjective distortions in beliefs $\Phi _0$ is always smaller than under certainty, as represented in (3), which creates an incentive to simply remain uninformed and choose the self-serving option. This strategy, however, represents a sort of self-deception which, in turn, is likely to be associated with a feeling of displeasure with one’s own self-serving rationalization. To take this into account, we again follow Rabin (1994) and the subsequent literature by introducing costs of self-deception $\Psi (\cdot )$ which increase with the misperception of probability $\mu$ and dependent on the amount of available information revealed, i.e., the value of *k*. The costs of self-deception for *k* signals revealed are

$$\begin{aligned} \Psi _k=\Psi _k \left( (\hat{\mu }_k-\mu _k), k;\beta \right) . \end{aligned}$$

A higher $\beta$ represents a greater sensitivity to self-deception, which varies across individuals as well as with contextual variables.

Given these assumptions and for *k* signals revealed, the valuation of the self-serving option *s* is

4 $$\begin{aligned} U_k(s)=m-c_s-\Phi _k\left( \hat{E}(d \left| k\right) -\hat{E}(\hat{\omega }_s\left| k\right) \right) -\Psi _k \left( (\hat{\mu }_k-\mu _k), k\right) . \end{aligned}$$

Note that for the signal revealing the truth with certainty, as assumed here, the case for $k=1$ reduces to (1). Using this setup, we can now proceed to analyze the tendency to avoid information in individual choices under hidden information. To identify the potential for self-serving information avoidance, consider an individual with green preferences ($\Phi _0>0$) for whom, under certainty, (3) holds, i.e., under certainty they would want to choose the green option, even if it is associated with higher costs $c_{\lnot s}$. For simplicity, we assume risk-neutrality. This individual’s expected valuation when planning to reveal the information, but before doing so, is

5 $$\begin{aligned} EU_{k=1}=m- \left[ \hat{\mu } \cdot c_s+(1-\hat{\mu })\cdot c_{\lnot s} \right] . \end{aligned}$$

The term subtracted from the endowment *m* represents the expected cost before the signal is revealed. In this case, given that (3) holds, the individual will only choose the self-serving option if interests are aligned; otherwise, option $\lnot s$ is purchased. As the individual intends to always choose the “greener” option, no cost from cognitive dissonance will arise.

For such an individual, the decision to remain uninformed is determined by a comparison of (4) with k=0 and (5). More precisely, self-serving information avoidance will arise iff $$ U_0(s)> EU_{k=1}$$, which is the case for

$$\begin{aligned} \Delta _c>\frac{\Psi _0+\Phi _0}{1-\mu }. \end{aligned}$$

Let us assume, for ease of presentation, that the cognitive dissonance is completely resolved if the voter remains uninformed, i.e., $\Phi _0=0$. Consequently, information will be avoided if, in the uninformed state, the costs of self-deception are not too high compared with the difference in costs. Taking also condition (3) into account, we can establish a price range, where such information avoidance is self-serving. Information avoidance leads to the exploitation of moral wiggle room iff

6 $$\begin{aligned} \Phi (d-\omega _s)> \Delta _c>\frac{\Psi _0}{1-\mu }. \end{aligned}$$

We can thus identify self-serving information avoidance via a comparison of self-serving choices in the full information treatment with those in the treatments where this information can be actively revealed: For a certain range of cost differences, decision-makers would choose the non-selfish option under full information, but remain uninformed and choose selfishly under hidden information. As with increasing differences in costs, condition (3) is increasingly less likely to hold, we can also expect an increasing share of self-serving choices under full information. Thus, for the parameterization chosen here, we expect information avoidance to be more likely to occur for our lower payoff differences. This is in line with recent experimental evidence on information avoidance in consumption. Momsen and Ohndorf (2022) report that for information structures with stochastic revelation, self-serving information avoidance is more likely to arise if the differences in payoffs are not too large. A similar effect is reported in Momsen and Ohndorf (2020) for complete revelation and small but positive information costs.

### Voting under full information

In order to predict the effects for our voting treatments, we present the situation in our group treatments as a Bayesian voting game with symmetric strategies, as in Feddersen and Pesendorfer (1997) and Tyson (2016), where equilibrium strategies are determined via cutoff levels. Notice that our treatments did not allow for abstentions; hence, for the case of complete information, the voter’s choice is determined exclusively via a comparison of expected utilities from voting for options *s* and$\lnot s$. More precisely, we follow Feddersen and Pesendorfer (1997) and Tyson (2016) by determining the cutoff levels in the (subjective or objective) distribution of voter preferences at which a specific voter would switch from voting for option $\lnot s$ to option *s* .

To represent our voting treatments, let there be $n + 1$ voters indexed by $j\in \{1,\ldots , n + 1\}$. As decisions are not only taken for the individual, but for the whole group, we introduce an additional utility component, denoted by $\nu _j$, which arises if the green option is chosen for the whole group. Thus, in contrast to the considerations by Brennan and Lomasky (1993), an individual choice framed as a consumption decision might not be considered an appropriate baseline treatment. We therefore included the dictatorship treatment to provide an additional point of comparison with our voting treatments.

As we are interested in moral biases, our analysis focuses exclusively on situations with conflicting interests, which is also standard when considering moral wiggle room (Dana et al., 2007). Thus, in this case, situation $\lnot s$ will be the greener option. The option that is chosen for the whole group is determined via simple majority voting. Thus, if the number of votes for option *s* is larger or equal to $(n/2+1)$, option *s* will be the outcome for all voters, otherwise $\lnot s$ will be chosen. Again, note that abstentions are impossible here, such that voter *j*’s strategy space is simply $S=\{\lnot s_j; s_j\}$, i.e., the only decision to be made is whether to vote for one option or the other.

We denote with $\pi _t$ the (subjective) probability of the individual being pivotal, and with $\pi _s$ ($\pi _{\lnot s}$) the (subjective) probability of the low (high) cost option being chosen if the player is not pivotal. In a conflicting interest situation under full information, voter *j*’s expected payoff when casting a vote for option $\lnot s$ is

7 $$\begin{aligned} U_{\lnot s}^v=m+\pi _t \left( \nu _j-c_{\lnot s}\right) +\pi _{\lnot s} \left( \nu _j-c_{\lnot s}\right) -\pi _s c_s, \end{aligned}$$

while voting for option *s* yields

8 $$\begin{aligned} U_{ s}^v=m-\pi _t c_s+ \pi _{\lnot s} \left( \nu _j-c_{\lnot s}\right) -\pi _s c_s-\Phi ^v_j \end{aligned}$$

Notice here that the additional utility component $\nu _j$ always arises if the group decision is in favor of option $\lnot s$ independently of whether the vote of individual *j* is decisive. Hence, $\nu _j$ is not interpreted as some sort of imperfect altruism, but represents a form of instrumental utility from choosing the greener option. Note that the total amount of contributions to offsets will increase with group size, as choosing $\lnot s$ will increase the contribution to the offset for every individual in the group. Hence, as pointed out by Aldrich (1993) and Myatt (2015), the decrease in expected instrumental utility with increasing *n* is likely to be lower than that modeled in Brennan and Lomasky (1993). Note that this effect would strengthen the prediction made by the low-cost theory of voting that larger *n* would increase the likelihood of more moral outcomes, as not only the relative weight of the expressive utility component increases with *n*, but also instrumental utility might change in favor of the greener option $\lnot s$.

Note also that, within (7) and (8), we continue to assume $\Phi ^v_j=\Phi (d_j-\omega _i;\alpha _j, n)$ to be the costs of dissonance, with $\Phi (0)=0$, $\Phi '>0$, $\Phi ''>0$, and $d_j\in \{\omega _s; \omega _{\lnot s}\}$. Parameter $\alpha$ represents the intensity of the green preference, with $\frac{d \Phi }{d\alpha }>0$. We further assume that $\alpha \in [0, \bar{\alpha }]$ is distributed over the voter population with probability distribution $F_\alpha$. The single voter knows their own preference level and the distribution *F* over the entire population.

Following Feddersen and Pesendorfer (1997), we denote a pure strategy for voter *j* as $\rho _j$, which is a measurable function from her preference type $\alpha$ to a vote choice, i.e., $\rho _j : \mathbb {R} \rightarrow S$ and a mixed strategy $\bar{\rho }_j$ is a measurable function from a voter’s type $\alpha$ to the probability of voting for option *s*, i.e., $\bar{\rho }_j : \mathbb {R} \rightarrow [0,1]$.

Let us denote with $\hat{\alpha }$ the cutoff preference level for which $U_{\lnot s}^v=U_{ s}^v$ [as in (7) and (8)], i.e.,

9 $$\begin{aligned} \Phi ^v(\hat{\alpha })=\pi _t \ (\Delta _c- \nu ). \end{aligned}$$

Thus, $\hat{\alpha }$ represents the preference type that is indifferent between choosing *s* and $\lnot s$. As under full information this is the only choice to be made, the formulation of the voter’s strategy is almost trivial. Depending on their preference type $\alpha$, the voter will choose the option that maximizes their utility. Hence, as abstentions are impossible, the voter’s dominant strategy for any $\hat{\alpha }$ is

10 $$\begin{aligned} \bar{\rho }_j(\alpha _j)={\left\{ \begin{array}{ll}1 \text { for } \alpha _j<\hat{\alpha } \\ 0 \text { for } \alpha _j>\hat{\alpha }\\ \frac{1}{2} \text { for } \alpha _j=\hat{\alpha } \end{array}\right. } \end{aligned}$$

Thus, for any distribution *f* over $\alpha$ (or a belief thereof), the probability *q* that a randomly selected voter votes for *s* under complete information is

11 $$\begin{aligned} q^{f} = \int _0^{\hat{\alpha }} \bar{\rho }_j(\alpha _j) \ f(\alpha ) \ \textrm{d} \alpha \end{aligned}$$

In other words, $q^{f}$ is the probability that any randomly drawn voter will choose the selfish option *s*. Hence, for the corresponding unique symmetric Nash equilibrium $\bar{\rho }^*$, the probability of being pivotal in our voting treatments is determined by the standard binomial distribution

12 $$\begin{aligned} \pi _t(q)=\left( {\begin{array}{c}n\\ n/2\end{array}}\right) \cdot q^{\frac{n}{2}}\cdot (1-q)^{\frac{n}{2}} \end{aligned}$$

where we substitute $q=q^{f}$.

Notice that for our Voting3 and Voting11 treatments, the initial expectations over *q* might differ among subjects, as they might have different beliefs over the distribution $f(\alpha )$. Yet, we can expect the following considerations to hold for aggregated decisions. Note further that for our Dictator treatment, the dominant strategy would also be determined by (9) and (10), while pivotality $\pi _t$ is fixed exogenously at 1/3. Thus, for all group treatments, it follows from (9) and (10) that a subject in a situation with conflicting interests will choose the greener option $\lnot s$ with certainty if and only if

13 $$\begin{aligned} \nu +\frac{\Phi ^v(\alpha _j)}{\pi _t}>\Delta _c, \end{aligned}$$

i.e., if the costs of cognitive dissonance divided by the probability of being pivotal and the additional utility if the green option is implemented for the entire group exceed the difference in costs between the selfish and the non-selfish option.

Several interesting observations can be made with respect to this result. First, notice that this condition is relaxed with a decrease in the probability of being pivotal $\pi _t$. Thus, with decreasing probability of the own vote being decisive, voting for option $\lnot s$ is more likely to be used as a strategy to avoid cognitive dissonance (cost $\Phi ^v$). If it is unlikely that voting for the “greener” option will affect the outcome, players can hence use the vote to align their choice with their own environmental ideals. The vote will thus reflect what the individual considers to be morally preferable, rather than being guided by their own narrow self-interest. This corresponds to the result derived from theories of expressive voting, where “moral” votes are likely to increase with larger constituencies. Thus, we formulate the following hypothesis:

#### Hypothesis 1 (a)

Under full information, the share of votes for the socially acceptable option in situations with conflicting interests increases in group size. This effect will be more pronounced for small payoff differences.

Notice that for n=10, which corresponds to our Voting11 treatment, the largest possible pivotality that can be derived from (12) is $\pi _t=0.25$, which would be the case for $q=1/2$. Hence, there exists no distribution of voter preferences *q* for which the probability of being pivotal $\pi _t$ is larger than 1/4.^16^ Consequently, for rational beliefs over *q*, the pivotality in Voting11 will always be smaller than for the Dictator treatment, which is exogenously set to 1/3. Thus, from (13), we formulate the following hypothesis for a comparison of behavior in a group with majority voting relative to a randomized dictatorship in situations with conflicting interests:

#### Hypothesis 1 (b)

Under full information, the share of votes for the socially acceptable option is larger in groups where the highest possible pivotality of a vote (Voting11) is smaller than the exogenously determined pivotality in the other group (Dictator). This effect is more pronounced for small payoff differences.

### Information avoidance in voting

Next, we consider voting decisions if players have the possibility to avoid information, as in our voting treatments with hidden information. In this case, we also consider the subjective probabilities for the outcome in the uninformed state. Thus, denote with $\pi _{t}^{u}$ the uninformed individual’s subjective probability of being pivotal, and with $\pi _{s}^{u}$ ($\pi _{\lnot s}^{u}$), the subjective probability of a majority for the low (high) cost option if the uninformed player is not pivotal. While these probabilities might be subject to individual over- or underestimation, we assume sufficient rationality in the sense that subjective probabilities add up to 1, i.e., $\pi _{s}^{u}+\pi _{\lnot s}^u+\pi _{t}^{u}=1$.

For ease of presentation, we again assume that the cognitive dissonance is completely resolved if the voter remains uninformed, i.e., $\Phi _0^v=0$, and only costs of self-deception $\Psi _0^v$ arise. Thus, analogous to (5) in the individual case, the voter’s expected payoff if information is avoided is as follows:

14 $$\begin{aligned} U_{k=0}^h(s)=m-\left( \pi _{\lnot s}^u c_{\lnot s}+ \pi _{s}^{u} c_s+\pi _{t}^{u} c_{s}\right) -\Psi _0^v(\beta ,k) \end{aligned}$$

In order to establish a cutoff preference level $\hat{\beta }$ separating informed and uninformed voters, this payoff needs to be compared with the ex ante expected payoff of a voter deciding to reveal information. Note that a voter with $\alpha <\hat{\alpha }$ would always choose the option *s*, as established above. As interests are aligned with probability $\mu$, an informed voter with $\alpha >\hat{\alpha }$ would vote for option $\lnot s$ in case of conflicting interests, i.e., her expected payoff before revealing information is

15 $$\begin{aligned} EU_{k=1}^h= m- \mu c_s - (1-\mu ) \left[ \pi _t \left( -\nu +c_{\lnot s}\right) +\pi _{\lnot s} \left( -\nu +c_{\lnot s}\right) +\pi _s c_s\right] . \end{aligned}$$

Thus, there potentially exist two different cutoff levels for preference parameters $\alpha$ and $\beta$, which are distributed with distribution function $F(\alpha , \beta )$.

We define the cutoff preference level $\hat{\beta }$ as the value of $\beta$ for which $U_{k=0}^v(s)= EU_{k=1}^v$, i.e., the preference level for which an individual is indifferent between revelation and non-revelation of information. For ease of presentation, we assume that at the margin, the voter will vote for the self-serving option *s*. In this case, and for $\hat{\alpha }$ as determined in (9), the dominant strategy for any voter is

16 $$\begin{aligned} \bar{\rho }_j(\alpha _j, \beta _j)={\left\{ \begin{array}{ll}1 \text { for } \beta _j \le \hat{\beta } \\ 1 \text { for } \beta _j>\hat{\beta } \text { and } \alpha _j \le \hat{\alpha }\\ 0 \text { for } \beta _j>\hat{\beta } \text { and } \alpha _j>\hat{\alpha } \end{array}\right. } \end{aligned}$$

The probability of any individual choosing the selfish option in any type of situation is then

17 $$\begin{aligned} q^{H} = F_{\alpha }(\hat{\alpha })\ \bar{\rho }(\alpha ) + F_{\beta } (\hat{\beta })\ \bar{\rho }(\beta ) - \int _0^{\hat{\alpha }}\int _0^{\hat{\beta }} \bar{\rho }(\alpha , \beta , k)\ f(\alpha ,\beta ) \ \textrm{d} \alpha \ \textrm{d} \beta \end{aligned}$$

Note that for a situation with aligned interests, both informed and uninformed voters would choose option *s*. Thus, an uninformed voter who observes $\lnot s$ being chosen can infer that $\lnot s$ must be the “greener” option (i.e., the situation features a conflict of interests), as it generated a positive number of votes. Thus, we can reformulate (14) as follows

18 $$\begin{aligned} U_{k=0}^h(s)=m- \mu c_s - (1-\mu ) \left( \pi _{\lnot s} (c_{\lnot s}-\nu ) + \pi _{s} c_s+\pi _{t} c_{s}\right) -\Psi _0^v \end{aligned}$$

The cutoff level $\hat{\beta }$ is hence determined via equality of (15) and (18), which leads to the following implicit definition:

19 $$\begin{aligned} \psi (\hat{\beta }) = (1-\mu ) \pi _t(q^h) \left( \Delta _c-\nu \right) \end{aligned}$$

Note that, again, $\pi _t(q^h)$ is determined analogously to the full information case via substitution of (17) in (12) for our voting treatments, while it is exogenously set to 1/3 in our Dictator treatment.

Exploitation of moral wiggle room via information avoidance arises if voters remain uninformed and vote for the self-serving option *s* who would have chosen $\lnot s$ under full information. As information costs are zero, we can thus, using (13), (16), and (19), establish the following condition for which a voter *j* exploits moral wiggle room via self-serving information avoidance:

20 $$\begin{aligned} \nu +\frac{\Phi ^v(\alpha _j)}{\pi _t(q^f)}>\Delta _c>\nu +\frac{\Psi _0^v(\beta _j)}{(1-\mu ) \ \pi _t(q^h)}. \end{aligned}$$

This result is particularly interesting. If (20) holds, expressive voting is no longer the voter’s preferred strategy to resolve the internal conflict between self-interest and environmental ideals. Instead, the player’s corresponding cognitive dissonance is managed via the avoidance of information on the effects of their own choice. These subjects with green preferences of intermediate intensity will remain uninformed and choose the self-serving option, even if their choice had been the opposite under full information.

As shown in the mathematical appendix, for an increasing number of voters *n*, the left-hand side in (20) increases at a higher pace than the right-hand side. This implies that if a constituency becomes larger, the range of payoffs for which information avoidance is the dominant strategy increases. Hence, while we expect a larger number of “green” choices in the Voting11 treatment under full information than in the corresponding Voting3 treatment due to expressive voting, this difference should be significantly lower for the respective treatments with hidden information. In other words, it can be expected that a larger share of voters will substitute expressive voting with information avoidance in Voting11 than in Voting3, as it is the dominant strategy.

Furthermore, the upper boundary of (20) is more likely to be exceeded for our larger price differences. Hence, again, self-serving information avoidance can be expected to be more likely to occur for our lower price differences. We summarize these predictions in the following hypothesis:

#### Hypothesis 2 (a)

The difference in the share of votes for the self-serving option between full and hidden information is larger in groups with more voters.

As laid out above, for rational beliefs, the largest possible pivotality in our Voting11 treatment is $\pi _t=1/4<1/3$. In the mathematical appendix, we show that if beliefs are rational (or at least the formation thereof), the above-made considerations also hold for the comparison of our Voting11 and Dictator treatments. We can hence formulate an analogous hypothesis:

#### Hypothesis 2 (b)

The difference in the share of votes for the self-serving option between full and hidden information is larger in groups where the highest possible pivotality of a vote (Voting11) is smaller than the exogenously determined pivotality in the other group (Dictator).

## Results

In our analysis of the experimental results, we will first focus on the effect of the different decision contexts on the share of selfish choices under full information (Hypothesis 1). We will then examine behavior under hidden information and explore the exploitation of moral wiggle room within each decision context (Hypothesis 2). Summary statistics for our sample, as well as power calculations, are provided in the Supplementary Material (see Table A.2 and Sect. A.4.2).

### Full information

To study the share of selfish choices across treatments, we only consider decision situations with conflicting interests, i.e., situations where the payoff-dominated option is associated with larger positive externalities than the payoff-optimal option. This leaves us with approximately half of the total observations for each treatment condition. Table 2 lists the share of selfish choices in decision situations with conflicting interests in the treatments with full information, pooled over all payoff differences in the left panel, for decision situations with payoff differences of 5 or 10 ECUs in the middle panel, and for decision situations with payoff differences of 15 or 20 ECUs in the right panel.^17^ Note that subjects in the treatments with full information are immediately aware of the conflicts of interest regarding their own monetary payoff and the associated contribution to carbon offsets.

In our full information treatments, choices appear to differ with respect to the decision contexts, as the shares of selfish choices reported in Table 2 suggest. These differences are more pronounced if the differences in payoffs between the two allocations are relatively small. For both, the aggregated data and for situations with a relatively small difference in payoffs, the share of selfish choices is lowest in the individual decision framed as a consumption choice and highest in the Voting3 treatment. Thus, the considerations in Brennan and Lomasky (1993) which juxtapose markets to democratic decisions, with the former leading to less moral outcomes than the latter, do not seem to be confirmed here. However, we seem to find some confirmation for the low-cost theory of voting as stated in Brennan and Lomasky (1993; as stated in Hypothesis 1a), for low payoff differences the share of selfish choices is indeed unambiguously smaller for Voting11 than for Voting3. The same holds if we compare Voting11 and the Dictator treatment (Hypothesis 1b).

As the low-cost theory of voting hinges on differences in pivotalities, note that for rational beliefs, the probability of being pivotal in Voting11 is strictly smaller than that in Voting3. Using equation (12), we therefore compute the theoretical pivotalities $\pi _t$ for our treatments, which would result from perfect anticipation of the actually observed shares of selfish votes. Since we implemented decision situations that varied in payoffs and payoff differences, we report those values for $\pi _t$ in Table 2 that correspond to the average of the pivotalities for each decision situation. The empirical shares of observed situations where one voter was decisive reflect the difference in pivotalities very well. For Voting3, this share was 0.378, while for Voting11 it is significantly lower, at 0.125.^18^

**Table 2:** Share of selfish choices in conflict situations under full information

	All data			Payoff diff. $\le$ 10			Payoff diff. > 10		
Treatment	$\hbox {N}^a$	Share of selfish choices	$\pi _t$	*N*	Share of selfish choices	$\pi _t$	*N*	Share of selfish choices	$\pi _t$
Ind. choice	576	0.561		276	0.377		300	0.730	
Voting3	576	0.639	0.421	258	0.566	0.461	318	0.698	0.391
Dictator	576	0.594	1/3	276	0.486	1/3	300	0.693	1/3
Voting11	528	0.612	0.102	220	0.405	0.139	308	0.760	0.076

$^a\hbox {N}$ refers to the total number of conflict situations for each treatment. It depends both on the number of participants per treatment and on the number of decision situations falling into each category

To further investigate these preliminary observations, we run several random-effects panel regressions with standard errors clustered on the subject level.^19^ The results for the pairwise comparisons of the institutional settings under full information are presented in Table 3.^20^ We regress a dummy variable indicating whether a choice was selfish on the period number to capture potential time trends, as well as on the squared period number to capture non-linear trends. We also include a dummy variable which indicates whether the difference in payoffs was high (HighPD = 1 if the difference in payoffs was 15 or 20 ECUs).^21^ Our main focus lies on the dummy variables for pairwise treatment comparisons. Furthermore, we interact these dummy variables with the dummy for the difference in payoffs between the options to control for the possibility of payoff differences having different effects across treatments. We include a rather lean set of control variables consisting of the subject’s age and whether they identified as male, as well as a dummy variable capturing whether they studied economics or a closely related subject.^22^ The latter is included to control for the influence of training in economics on decisions made in situations that resemble dictator and public good games.

**Table 3:** Selfish choices under full information across treatments

	(1)	(2)	(3)	(4)	(5)	(6)
	I versus D	I versus V3	I versus V11	D versus V3	D versus V11	V3 versus V11
Period	0.009	0.013*	0.021***	0.014**	0.022***	0.026***
	(0.007)	(0.007)	(0.006)	(0.006)	(0.006)	(0.006)
Period2	−0.000	−0.000	−0.000**	−0.000	−0.000**	−0.000**
	(0.000)	(0.000)	(0.000)	(0.000)	(0.000)	(0.000)
HighPD	0.370***	0.363***	0.344***	0.192***	0.174***	0.142***
	(0.045)	(0.045)	(0.045)	(0.039)	(0.039)	(0.039)
Dictator	0.136*					
	(0.073)					
Dictator*HighPD	−0.170***					
	(0.059)					
Voting3		0.158**		0.040		
		(0.079)		(0.081)		
Voting3*HighPD		−0.207***		−0.035		
		(0.061)		(0.057)		
Voting11			0.027		−0.127*	−0.170**
			(0.071)		(0.073)	(0.077)
Voting11*HighPD			−0.018		0.150***	0.178***
			(0.060)		(0.056)	(0.056)
Age	−0.010	0.014	−0.009	0.024**	0.003	0.016
	(0.011)	(0.013)	(0.006)	(0.010)	(0.008)	(0.013)
Male	0.101	0.014	0.099	0.103	0.217***	0.103
	(0.067)	(0.068)	(0.060)	(0.077)	(0.065)	(0.070)
Econ	0.042	0.128*	−0.016	0.079	−0.090	0.030
	(0.071)	(0.066)	(0.062)	(0.076)	(0.068)	(0.067)
Constant	0.467*	−0.074	0.356**	−0.180	0.200	−0.062
	(0.248)	(0.307)	(0.147)	(0.237)	(0.177)	(0.297)
*R*$^2$	0.104	0.106	0.163	0.083	0.151	0.136
*N*	1152	1152	1104	1152	1104	1104

Output from random-effects panel regressions. Standard errors (in parentheses) are clustered on the subject level. The dependent variable is a dummy which takes a value of 1 if the selfish option is chosen in a conflict situation. Male is a dummy variable which takes a value of 1 if the subjects identified as male. Econ is a dummy variable that takes a value of 1 if the subject studies economics or business. $$^{*}$$$$p<0.10$$, $$^{**}$$$$p<0.05$$, $$^{***}$$$$p<0.01$$

In all specifications, a higher difference in payoffs has a significant positive impact on the number of selfish choices, i.e., if it is more expensive to realize a higher climate benefit, fewer subjects decide to do so. Furthermore, the selfishness of choices tends to increase slightly over time.

As to the treatment comparisons, our initial observations are confirmed. There are indeed significantly more selfish choices in the Dictator and Voting3 conditions than in the individual choice setting, although in both cases this effect disappears for larger payoff differences.^23^ Notice also that there is no significant difference in selfish choices when comparing individual choices to votes in Voting11 (Regression 3). Thus, when moving from individual to group decisions, the number of green choices first decreases and then approaches the level achieved in the individual choice context if the size of the constituency increases. This seems to indicate that decisions in and for groups also reflect additional other-regarding preferences. A decision reduces the payoff not only of the individual themselves, but also of the other group members. Thus, we conclude that, in contrast to the initial considerations made by Brennan and Lomasky (1993), a market situation might not represent a useful baseline to which group decisions with voting should be compared.

A closer look at the regression results for our group treatments reveals that a difference in the size of the constituency does indeed have an effect on the number of green decisions. For smaller price differences, the share of selfish choices in Voting11 is significantly lower than that in Voting3 (Regression 6). The same seems to hold when comparing Voting11 and the Dictator treatment (Regression 5). For larger price differences, this effect disappears. This confirms our Hypothesis 1, which predicts an increased incentive for expressive voting for Voting11 if the corresponding sacrifice in own payoff is small enough. This is also in line with results reported in Tyran (2004) where, in a different setup, higher instrumental costs reduce the tendency for expressive voting.

A comparison of the regression results for Voting3 and the Randomized Dictator setting reveals that the corresponding numbers of green choices are statistically indistinguishable (see Regression 4) for low differences in payoffs.^24^ This deserves some discussion. Note that the differences in $\pi _t$ as reported in Table 2 are not too large. Furthermore, some subjects in the Dictator treatment may have interpreted the decision from the stance of already having been chosen as a dictator. In this case, their perceived probability of being pivotal would be 1. Others, in contrast, may perceive their pivotality as the actual $\frac{1}{3}$. Hence, on average, the perceived pivotality may be comparable to that of Voting3. If this were the case, expressive voting would be robust to the framing of the decision.^25^ As a consequence, our results with respect to framing remain inconclusive.

### Hidden information and exploitation of moral wiggle room

When considering the treatments with hidden information, differences in the share of selfish choices are less pronounced than under full information. As a comparison of the share of selfish choices listed in Table 4 indicates, differences in the number of voters or in pivotality no longer seem to play a role in the choice of allocations if information is initially hidden but revealable.^26^ This indicates that our Hypothesis 2 is indeed supported by our data. Note that, again, the order of theoretical pivotalities is supported by the share of situations where one voter was decisive. This share was 0.457 for Voting3 and significantly lower at 0.104 for Voting11 (see Table 8 in the Appendix). Still, the share of selfish choices seems to be unaffected by differences in pivotality.

To further investigate this observation, we perform a regression analysis for the hidden information treatments analogous to the full information case, the results of which are reported in Table 5.^27^ While both the effect of the period number and of the difference in payoffs are similar to the full information treatments, none of the dummy variables specifying the decision context has a significant impact on the number of selfish choices in conflict situations. Hence, the possibility of avoiding information seems to indeed negate any effect that the decision context has on moral choices under full information.

**Table 4:** Share of selfish choices in conflict situations under hidden but revealable information

Treatment	All data			Payoff diff. $\le$ 10			Payoff diff. > 10		
	*N*$^a$	Share of selfish choices	$\pi _t$	*N*	Share of selfish choices	$\pi _t$	*N*	Share of selfish choices	$\pi _t$
Ind. choice	576	0.696		276	0.616		300	0.770	
Voting3	576	0.734	0.376	258	0.659	0.424	318	0.796	0.341
Dictator	576	0.722	1/3	264	0.633	1/3	312	0.798	1/3
Voting11	528	0.759	0.065	220	0.645	0.105	308	0.841	0.037

$^a$N refers to the total number of conflict situations for each treatment. It depends both on the number of participants per treatment and on the number of decision situations falling into each category

**Table 5:** Selfish choices under hidden information across treatments

	(1)	(2)	(3)	(4)	(5)	(6)
	I versus D	I versus V3	I versus V11	D versus V3	D versus V11	V3 versus V11
Period	0.010	−0.002	0.012**	0.006	0.020***	0.009
	(0.006)	(0.005)	(0.006)	(0.006)	(0.006)	(0.006)
Period2	−0.000	0.000	−0.000	−0.000	−0.000*	−0.000
	(0.000)	(0.000)	(0.000)	(0.000)	(0.000)	(0.000)
HighPD	0.138***	0.143***	0.125***	0.194***	0.187***	0.138***
	(0.034)	(0.034)	(0.034)	(0.035)	(0.035)	(0.043)
Dictator	−0.042					
	(0.070)					
Dictator*HighPD	0.055					
	(0.049)					
Voting3		−0.038		0.054		
		(0.071)		(0.079)		
Voting3*HighPD		0.006		−0.046		
		(0.055)		(0.056)		
Voting11			−0.000		0.078	0.027
			(0.062)		(0.073)	(0.068)
Voting11*HighPD			0.043		−0.017	0.028
			(0.053)		(0.053)	(0.060)
Age	0.007	−0.009	−0.005	0.011	0.015	−0.003
	(0.008)	(0.009)	(0.008)	(0.012)	(0.010)	(0.012)
Male	0.052	0.157**	0.089	0.091	0.044	0.133**
	(0.065)	(0.064)	(0.059)	(0.062)	(0.057)	(0.059)
Econ	0.074	0.103*	0.096*	0.057	0.058	0.066
	(0.060)	(0.057)	(0.053)	(0.062)	(0.060)	(0.052)
Constant	0.374*	0.755***	0.567***	0.218	0.051	0.507**
	(0.195)	(0.203)	(0.190)	(0.290)	(0.270)	(0.255)
*R*$^2$	0.049	0.075	0.080	0.056	0.082	0.086
*N*	1152	1152	1104	1152	1104	1104

Output from random-effects panel regressions. Standard errors (in parentheses) are clustered on the subject level. The dependent variable is a dummy which takes a value of 1 if the selfish option is chosen in a conflict situation. Male is a dummy variable which takes a value of 1 if the subjects identified as male. Econ is a dummy variable that takes a values of 1 if the subject studies economics or business. $$^{*}$$$$p<0.10$$, $$^{**}$$$$p<0.05$$, $$^{***}$$$$p<0.01$$

#### Self-serving information avoidance or inattention?

**Fig. 1:**
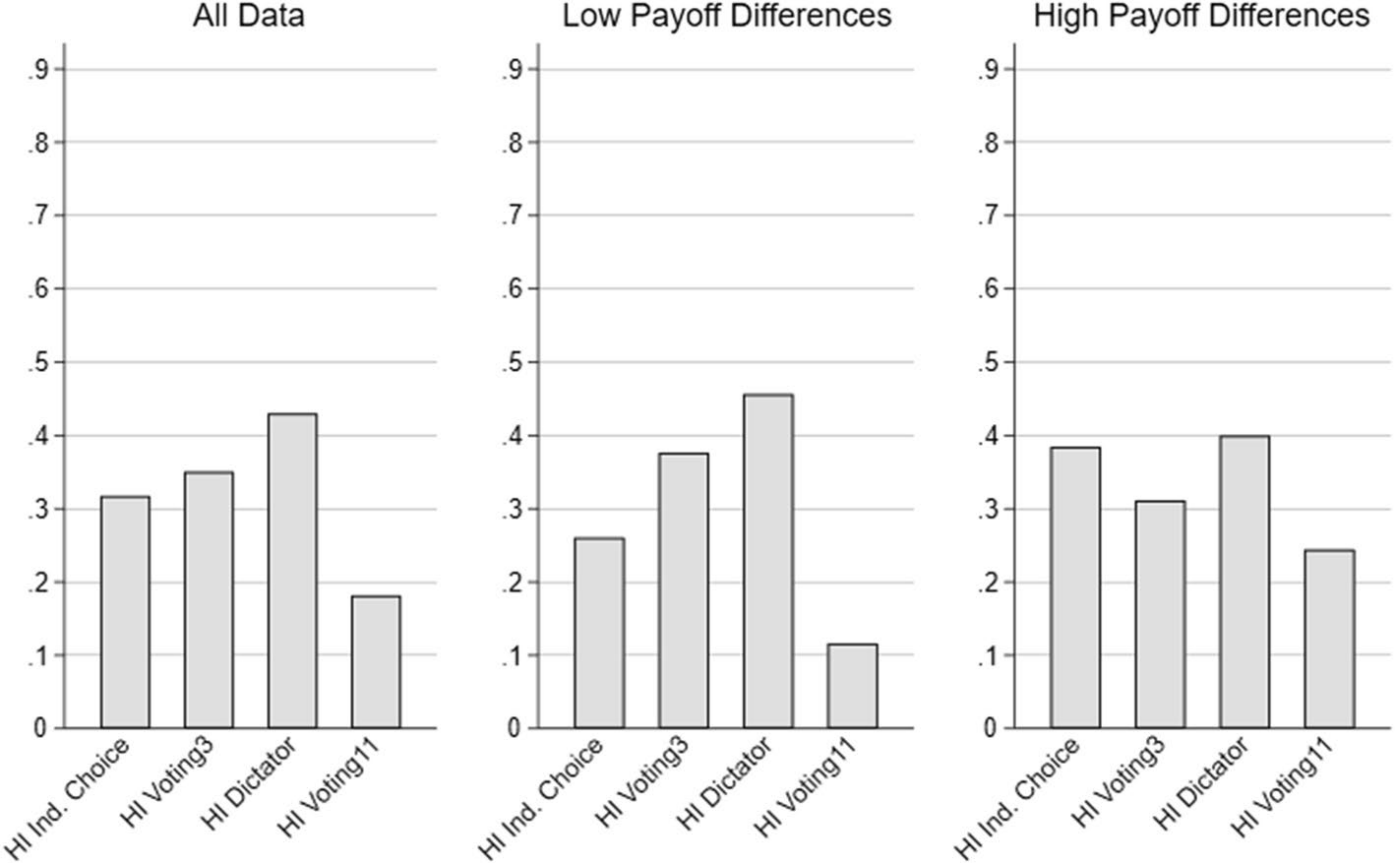
Share of selfish choices in conflict situations under hidden information (HI)—*informed* subjects

When considering only the choices of *willingly* informed subjects in the hidden information treatments, we observe roughly the same patterns as in the treatments with full information, yet at a lower level of selfishness (see Fig. 1). More precisely, the share of selfish choices is lowest in Voting11 and second lowest in the individual choice treatment. In the remaining two treatments, choices are significantly more selfish than in Voting11, with no significant difference between the Dictator and the Voting3 treatment. Thus, for those subjects who decided to reveal information, we find a significant amount of expressive voting, which is in line with our theoretical considerations.

**Fig. 2:**
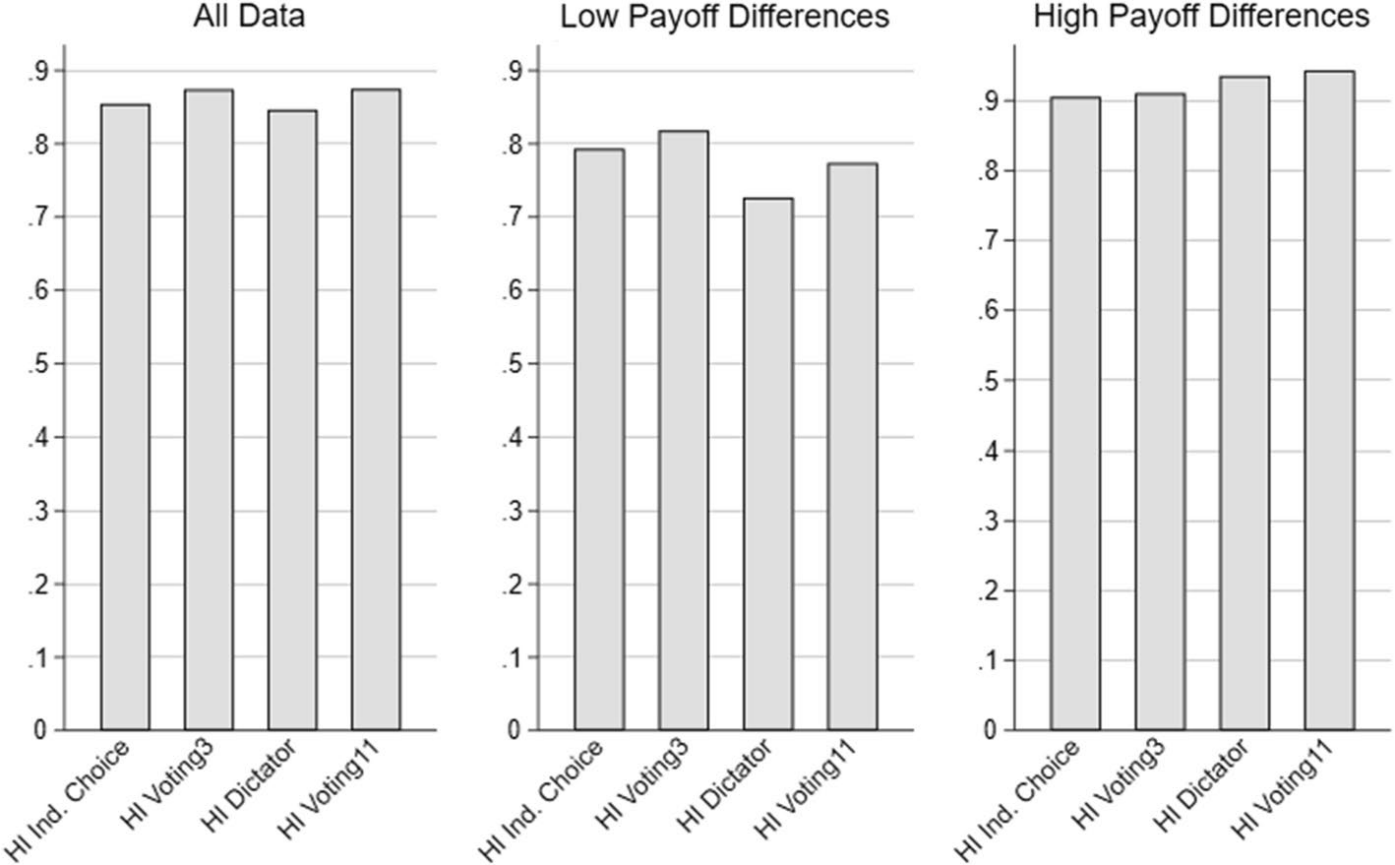
Share of selfish choices in conflict situations under hidden information (HI)—*uninformed* subjects

A closer look at the revelation decisions is also useful to check if our results can be better explained by cognitive costs of attention (Exley & Judd, 2021). We do indeed find some evidence for inattention. As shown in Fig. 2, the share of uninformed selfish choices does not correspond to 100% which would be expected if choices were perfectly rational. However, our results are inconsistent with inattention as the primary explanation for information avoidance. As shown in Fig. 1, the share of willingly informed subjects that ultimately choose the self-serving option in situations with conflicting interests varies between 18.2% (Voting11) and 43.0% (Dictator). We refer to this group as “curious egoists.” Note that if information revelation were associated with costs, even if only in the cognitive sense, an otherwise rational agent who is predisposed to choosing the self-serving option in a situation with conflicting interests would not want to incur these costs. Thus, such large shares of curious egoists cannot be explained by ignorance due to cognitive costs. In contrast, our model can indeed account for their existence, as such individuals would want to avoid Rabin’s cost of self-deception ($\psi _0$) even if their environmental preferences are not strong enough to choose option $\lnot s$ when informed. Thus, our data seem consistent with information avoidance as a strategy to reduce cognitive dissonance, while they are inconsistent with inattention as an exclusive explanation for information avoidance.

#### Exploitation of moral wiggle room

We now turn to the comparison of self-serving choices over information conditions to investigate potential exploitation of moral wiggle room. By comparing the number of selfish choices in situations with conflicting interests in our full information and hidden information treatments, we can identify the effect of self-serving information avoidance. If the share of selfish choices is significantly higher under hidden information than under full information, this provides evidence for subjects exploiting moral wiggle room (Dana et al., 2007).

**Fig. 3:**
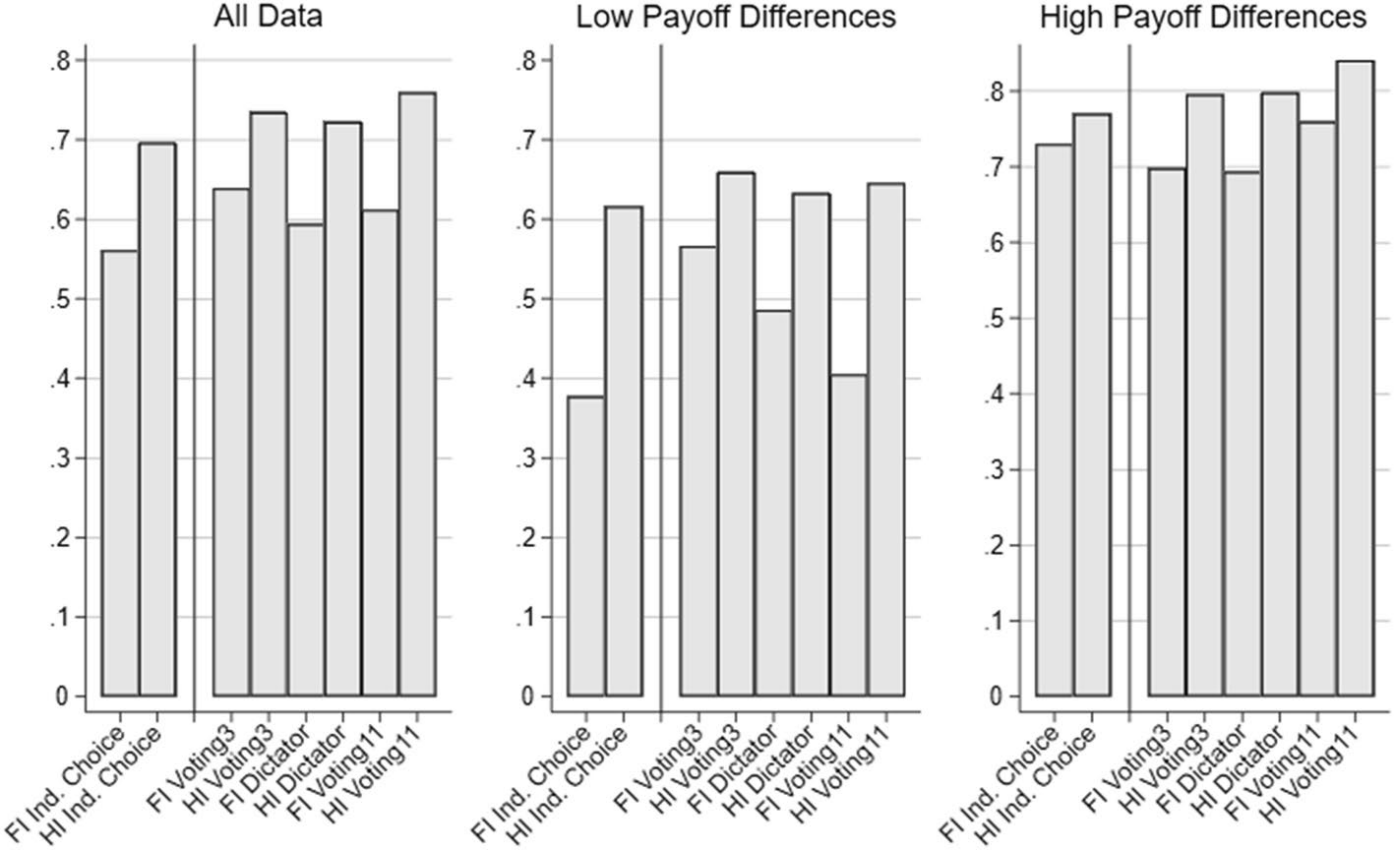
Share of selfish choices in conflict situations

The bars in Fig. 3 depict the average share of selfish choices in conflict situations for each decision context and information condition, either representing all data, situations with smaller payoff differences ($\Delta \le 10$), or larger payoff differences ($\Delta > 10$). Notice that in all institutional settings, the share of selfish choices is lower when externalities are initially observable (full information treatments), while the size of these differences seems to depend on the decision context. The difference in behavior is most notable in the individual choice setting and in Voting11. Again, as predicted, the effect of information avoidance seems to be more pronounced for smaller payoff differences. Thus, the graphs indicate that exploitation of moral wiggle room might indeed arise in the contexts investigated here.

To make more informed statements on the exploitation of moral wiggle room, we perform several regression analyses, the results of which are reported in Table 6. We regress the decision to choose selfishly in conflict situations on a dummy variable “Hidden” indicating whether the information on the associated offset level is initially unobservable. We also include the period number, the squared period number, a dummy indicating if the difference in payoffs was high, “HighPD,” and an interaction term of the payoff difference and the “hidden” information dummy as explanatory variables. Again, we control for the subject’s age, gender, and the field of study using the “Econ” dummy variable.^28^ As before, the results stem from random-effects panel regressions with standard errors clustered at the subject level.

**Table 6:** Selfish choices: moral wiggle room

	(1)	(2)	(3)	(4)
	I	V3	D	V11
Period	0.005	0.007	0.013**	0.029***
	(0.007)	(0.006)	(0.006)	(0.006)
Period2	−0.000	−0.000	−0.000*	−0.000**
	(0.000)	(0.000)	(0.000)	(0.000)
HighPD	0.370***	0.155***	0.195***	0.323***
	(0.046)	(0.040)	(0.039)	(0.039)
Hidden	0.271***	0.070	0.069	0.269***
	(0.065)	(0.077)	(0.080)	(0.065)
HighPD*Hidden	−0.226***	−0.010	−0.002	−0.154***
	(0.055)	(0.059)	(0.052)	(0.055)
Age	−0.016*	0.019	0.016*	0.000
	(0.009)	(0.015)	(0.009)	(0.007)
Male	0.061	0.092	0.111	0.142**
	(0.062)	(0.073)	(0.069)	(0.057)
Econ	0.115**	0.125*	0.002	−0.028
	(0.057)	(0.065)	(0.071)	(0.056)
Constant	0.629***	−0.026	0.019	0.070
	(0.211)	(0.330)	(0.221)	(0.167)
*R*$^2$	0.124	0.085	0.086	0.203
*N*	1152	1152	1152	1056

Output from random-effects panel regressions. Standard errors (in parentheses) are clustered on the subject level. The dependent variable is a dummy which takes a value of 1 if the selfish option is chosen in a conflict situation. Male is a dummy variable which takes a value of 1 if the subjects identified as male. Econ is a dummy variable that takes a value of 1 if the subject studies economics or business. $$^{*}$$$$p<0.10$$, $$^{**}$$$$p<0.05$$, $$^{***}$$$$p<0.01$$

A positive and significant coefficient for our Hidden dummy in Table 6 indicates that moral wiggle room was indeed exploited. Notice that the sign of the effect of the Hidden dummy on the number of selfish choices is always positive, but it is only highly significant for individual choices and in Voting11.^29^ For these two treatments, we can hence justifiably infer that moral wiggle room was exploited. Yet, as predicted, this effect is lower when the difference in payoffs is relatively large. In contrast, for the Dictator and Voting3 treatments, the exploitation of moral wiggle room did not occur in a significant manner.^30^ This is, in fact, not surprising, as for both treatments, the share of selfish choices was already particularly high under full information. Consequently, the additional effect of information avoidance cannot contribute significantly to the share of self-serving choices under hidden information.

Thus, while a comparison of both voting treatments under full information supported the low-cost theory of voting, exploitation of moral wiggle room seems to entirely counterbalance any effect of a change in pivotality under hidden information. Expressive voting no longer seems to be the preferred strategy for dealing with cognitive dissonance if information is initially unobservable. Instead, subjects exploit moral wiggle room via self-serving information avoidance in the Voting11 treatment. As suggested in our theoretical considerations, this effect is strongest for lower payoff differences.

#### Exploiting moral wiggle room in period 1

**Fig. 4:**
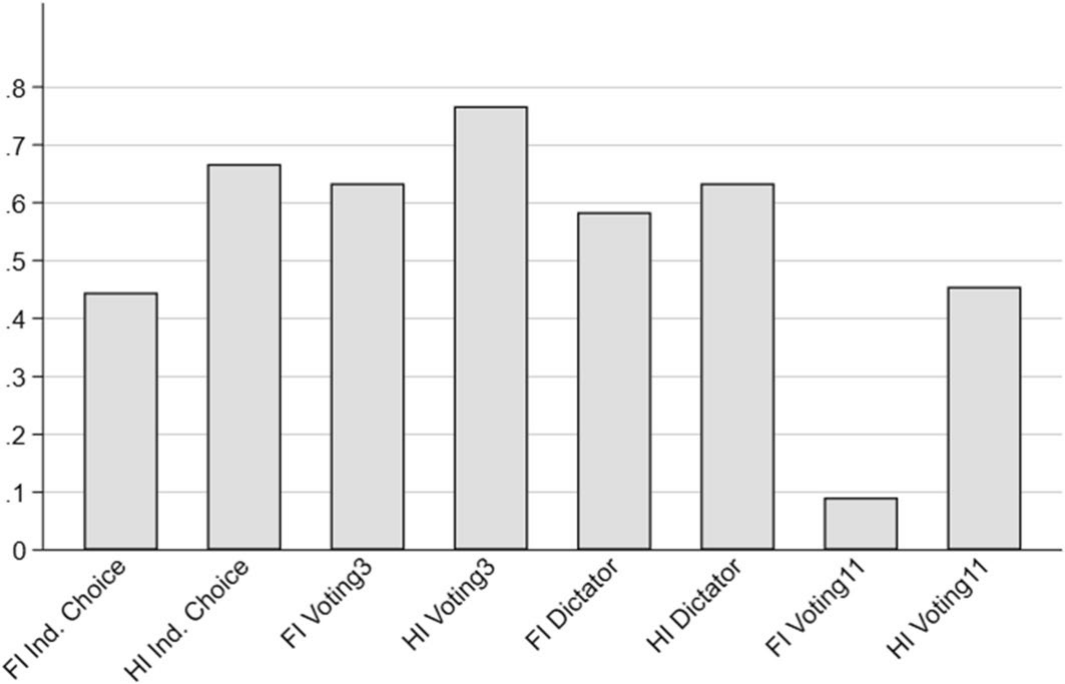
Share of selfish choices in period 1

To check whether behavioral patterns are significantly different in the first period when subjects did not yet know how the experiment would continue, we analyze first round-observations separately (see Table 7 for an overview and Fig. 4 for a graphical representation). In line with our findings when considering all 24 rounds, subjects exploit moral wiggle room in the individual choice condition and when voting in large groups. In addition, the share of selfish choices in immediately observable conflict situations is lowest for individual choices and in Voting11—both results supporting our findings for the full 24 rounds.

**Table 7:** Share of selfish choices in period 1

Treatment	Full information	Hidden information	*p*-value
	Share of selfish choices	Share of selfish choices	
Ind. choice	0.444	0.667	0.048
Voting3	0.633	0.767	0.199
Dictator	0.583	0.633	0.437
Voting11	0.091	0.455	0.008

*p*-values from Fisher’s exact tests

## Conclusion

We present the results of an incentivized laboratory experiment designed to investigate self-serving information avoidance and moral bias in individual and voting decisions. Subjects choose between two options which affect their own payoff and the amount contributed to carbon offsets such that the impact of the subjects’ decisions reaches beyond the laboratory. Our main interest lies in the resolution of potential cognitive dissonances between the subjects’ narrow self-interest and their self-perception as an environmentally conscious individual.

When information on the contribution is immediately observable, we observe the lowest share of self-serving decisions in the individual choice condition framed as consumption and with voting in large groups (Voting11), while the self-serving option is chosen more frequently if decisions are taken for a group of three, either by a randomized dictator (Dictator) or via voting (Voting3). This contrasts with the initial considerations by Brennan and Lomasky (1993) on expressive voting, who infer from their argument on pivotality that decisions on markets should be *less* moral than democratic decisions. However, we believe that this contrast needs to be qualified, as the two decision contexts differ with respect to factors besides pivotality. If decisions are taken for the whole group, additional factors are at play that influence the underlying trade-off between self-interest and environmental ideals. It is not unlikely that the difference between our individual choice condition and group decisions is due to the influence of distributive preferences, as a decision that is binding for the whole group also has an impact on the payoff of others. Thus, while the juxtaposition by Brennan and Lomasky (1993) might be normatively appealing, the market does not seem to be the most appropriate baseline to assess potential moral bias within democratic choice.

Comparing our voting treatments yields certainly the most interesting result for the full information case. An increase in the number of voters from 3 to 11 significantly increases the share of “greener” choices. This supports the low-cost theory of voting, as the corresponding decrease in the probability of being pivotal is associated with a decrease in self-serving choices. Hence, the relationship between the share of environmentally friendly decisions under full information and the number of decision-makers is not linear: The share of environmentally friendly decisions is high in individual decision-making contexts, decreases with majority voting in small groups, and increases again in large groups. Notice that there is no reason to assume that the effects causing the decrease in the share of environmentally friendly decisions in small groups disappear for larger groups, such that an isolated effect of the probability of being pivotal might even be larger than reported here.

When information on the externalities is initially hidden, but revealable, differences in self-serving choices between decision contexts disappear entirely, with all decisions being in general more selfish. Furthermore, in the settings where we observed the lowest share of selfish decisions under full information—in the individual choice condition and Voting11—subjects use the possibility of avoiding information as an excuse to behave more selfishly: They exploit moral wiggle room. In both decision contexts, remaining ignorant is used as a strategy to reduce cognitive dissonance, as it allows one to choose the self-serving option without being in open contradiction to environmental ideals. For Voting3 and the Dictator treatment, the observable pattern of self-serving ignorance is less pronounced, as choices are already more selfish under full information. Most interestingly, in the Voting11 treatment, information avoidance entirely substitutes expressive voting as a strategy to manage cognitive dissonance.

This latter result surely needs some discussion, as the idea of electoral choices being more moral is one of the most compelling results of the theory of expressive voting. The possibility of remaining uninformed with respect to the positions of candidates or parties concerning policies that feature a moral dimension is rather the rule than the exception.^31^ Moreover, in recent years, several phenomena have been identified supporting the conjecture that voters tend to actively avoid information. There seem to be, in fact, groups of voters that tend to self-select into echo chambers, isolating themselves from certain information sources.^32^ To the extent that this self-selection is an active choice, this development could also be interpreted as a more sophisticated form of information avoidance. As we have shown, the possibility of remaining uninformed can lead to a reversal of (revealed) voters’ preferences, as voters with intermediate preference intensity will remain uninformed and choose the option that serves their narrow self-interest. Hence, when it comes to actual elections, such behavior is likely to contribute to a polarization of voters’ preferences.

Note, however, that information avoidance not only provides an explanation for the polarization of preferences, but also could explain the polarization of politicians’ positions and policies themselves. In a Hotelling-Downs framework, policies will converge to the preferences of the median voter if utility functions are concave. In contrast, convex utility functions over an ordered policy range will lead to a polarization in politicians’ positions and their policies (Kamada & Kojima, 2014; Tajika, 2020). It is easy to see that a utility function that is concave over a specific policy range under complete information can become convex if voters remain uninformed with respect to a specific property of these options. In our experiment, for example, the set of policies was characterized by two dimensions, the associated cost and the corresponding effect on the global climate. If, under full information, concavity of utilities is driven by a decreasing marginal utility of environmental utility, self-serving avoidance of this information leads to an evaluation of these options with respect to cost only, for which a convex form is plausible.

Thus, in the context of climate policy, democratic elections will not necessarily lead to an increase in the stringency of mitigation efforts, as the theory of expressive voting would predict (Brennan, 2009). Indeed, this theory does not seem to adequately predict the political development in the political sphere of various countries, where we observe polarization of both voter preferences and proposed policy positions (Karakas & Devashish, 2020). As this discrepancy can be explained via self-serving information avoidance, it seems promising to further investigate these interactions in future research.

## Supplementary information

Below is the link to the electronic supplementary material.

Supplementary material 1 (PDF 534 kb)

## Footnotes

1 In social psychology, selective exposure has been examined in a vast number of experimental studies (Hart et al., 2009; Knobloch-Westerwick et al., 2017; Smith et al., 2008).

2 For a more detailed discussion of the two approaches, which were both initially conceived in social psychology, see Momsen and Ohndorf (2020). For a first formalization of cognitive dissonance to explain information avoidance in the context of individual decisions, see Momsen and Ohndorf (2022).

3 See, for example, Leviston et al. (2013), Bolin and Hamilton (2018), Walter et al. (2018), and Jasny et al. (2015, 2018).

4 An alternative representation of information avoidance, based on a dual-self model, has been laid out in Grossman and van der Weele, (2017). However, such a model is less suitable for integration into the low-cost theory of voting, as this would require two separate contracts between both selves.

5 This treatment also ensures comparability with previous lab experiments investigating moral wiggle room exploitation in individual decision-making. See, for example, Dana et al. (2007), Larson and Capra (2009), Fong and Oberholzer-Gee (2011), Matthey and Regner (2011), Grossman and van der Weele (2017), Feiler (2014), Grossman (2014), Lind et al. (2019), and Momsen and Ohndorf (2020, 2022).

6 This result also speaks to the growing literature on moral behavior in markets (see, e.g., Falk & Szech, 2013; Bartling et al., 2014; Pigors & Rockenbach, 2016; Sutter et al., 2016).

7 As pointed out in Tyran et al. (2019), moving from individual consumption to voting decisions has two different effects: a change in pivotality and a potential external effect on the payoff of others.

8 Note that expressive voting is not limited to the expression of moral principles. Robbett and Matthews (2018), for example, report robust evidence for voting as a means of expressing partisanship.

9 Tyran (2004) also finds that expressive voting can arise in low-cost situations but not in situations associated with higher (expected instrumental) cost.

10 This finding is also in line with the results presented in Momsen and Ohndorf (2020, 2022) where the interdependence between the difference in payoffs and information avoidance is analyzed in more detail.

11 Subjects received information about offsets before the experiment started. A translation can be found in the Supplementary Material (see Section A.3).

12 The main difference from the above-mentioned papers lies in the institutional settings that we implement in the study at hand. While, in previous papers, we only considered individual choices framed as consumption decisions, this paper investigates individual decisions as only one of the considered institutional settings among voting and randomized dictatorship (see below).

13 Note that this decision is structurally identical to a dictator game, where the decision-maker needs to decide between two allocations. Yet, the situation was framed as a consumption decision using terms such as “products,” “purchase,” and “price.”

14 In the Voting3 treatment, each session consisted of 24 subjects split into four fully independent matching groups, from which groups were randomly formed. Due to the large group size, this was not possible in the Voting11 condition.

15 Note that the provision of feedback does not seem to have influenced the exploitation of the moral wiggle room identified below. Our results for the first round, when subjects were unaware that they might receive feedback, exhibit very similar patterns as those from later rounds (see Sect. 4.2.3). Yet, even if feedback had an influence, the observed degree of willful ignorance would have to be interpreted as a lower bound for the actual exploitation of moral wiggle room in a situation without feedback.

16 The same logic is used to derive the predictions in Tyran (2004).

17 A choice is defined as selfish when a subject chooses the payoff-optimal option *s* that is associated with lower positive externalities than the payoff-dominated option. We use this definition regardless of the information condition. See Sect. A.4.8 in the Supplementary Material for a graphical representation of the shares of selfish choices.

18 The empirical frequencies of a voter being pivotal are hence similar to the theoretical values. We report these frequencies, the number of pivotal votes, and the effect of a pivotal vote on the outcome in Sect. A.4.1 in the Supplementary Material.

19 When we cluster standard errors on the matching group level, *p*-values are smaller. In Voting11 the number of matching groups is, by design, very low such that results may overstate the true levels of significance. We therefore report the results from regressions clustering standard errors on the subject level. As new groups were formed in each round and subjects remained anonymous, within-group correlation should be sufficiently low.

20 For non-parametric tests, see Sect. A.4.4 in the Supplementary Material.

21 In decision situations with *low* payoff differences (5 or 10 ECUs), efficiency-concerned decision-makers should prefer the option with the higher contribution to offsets. Facing *high* payoff differences, efficiency-concerned subjects should opt for the option associated with the higher individual payoff.

22 A regression analysis including a larger set of control variables can be found in Table A.7 in the Supplementary Material.

23 For the individual choices, the individual payoff was framed as endowment minus price. If this loss frame were to have an impact on subjects’ choices, decisions in the individual choice treatment should be *more* selfish than decisions in the other settings that employed a gain frame.

24 When using individual choices as baseline (see Table A.6 in the Supplementary Material), coefficients of the Dictator and the Voting3 dummy are also statistically indistinguishable (*p* = 0.602).

25 An alternative interpretation would be that the effect of pivotality is offset by some sort of responsibility diffusion. Yet, our evidence for such an interpretation is only indirect at best.

26 Non-parametric test results are reported in Sect. A.4.4 of the Supplementary Material.

27 The results of a regression analysis including a larger set of control variables are reported in Sect. A.4.7 of the Supplementary Material.

28 Regressions including a larger set of control variables are reported in Table A.8 in the Supplementary Material.

29 The non-parametric tests reported in Table A.4 in the Supplementary Material yield the same results for Voting11 and Individual Choice.

30 These results remain unchanged when considering only the decisions made in the first round (see Sect. 4.2.3).

31 Momsen and Markus (2022a) provide evidence that this effect is observable in the context of measures against COVID-19. In their experiment, voters of the Democratic Party seem to be prone to avoiding information at their own cost, while Republican voters rather avoid information in a self-serving fashion, as defined here.

32 See, for example, Sasahara et al. (2019), Pennycook and Rand (2019), Bakshy et al. (2015), Halberstam and Knight (2016).
