# Supplementary material for: Expressive voting versus information avoidance: experimental evidence in the context of climate change mitigation
Source: Public Choice. 2022 Dec 31;194(1-2):45–74. doi: 10.1007/s11127-022-01016-x (PMC9805350; doi:10.1007/s11127-022-01016-x)
Supplement: Supplementary file 1 — Supplementary material 1 (PDF 534 kb) [file 11127_2022_1016_MOESM1_ESM.pdf]

Supplementary Material to "Expressive Voting vs. Information Avoidance: Experimental Evidence in the Context of Climate Change Mitigation"

A.1 Mathematical Appendix

Proof of increasing range for information avoidance with increasing n

First note that information avoidance only exists if the left hand side of (20) (LH) is larger than the right hand side (RH), or if

$$\frac{\Phi^v(\alpha_j)}{\pi_t(q_c)} > \frac{\Psi_0^v(\beta_j)}{(1-\mu)\ \pi_t(q_h)} \text{ or } \frac{\Phi^v(\alpha_j)}{\Psi_0^v(\beta_j)} > \frac{\pi_t(q_c)}{(1-\mu)\ \pi_t(q_h)} \tag{A1}$$

Substituting (12) for π_t and simplifying yields

$$\frac{\Phi^v(\alpha_j)}{\Psi_0^v(\beta_j)} > \frac{(1-q_c)^{n/2} q_c^{n/2}}{(1-\mu)(1-q_h)^{n/2} q_h^{n/2}} \tag{A2}$$

Thus, in a voting treatment, information avoidance only exists if (A2) holds.
The range where information avoidance is possible will increase if $\frac{\partial LH}{\partial n} > \frac{\partial RH}{\partial n}$
This is the case if

$$\frac{\Phi^v(\alpha_j)}{\Psi_0^v(\beta_j)} > \frac{((1-q_h)q_h)^{-\frac{n}{2}}((1-q_c)q_c)^{n/2}\left(2H_{\frac{n}{2}} - 2H_n + \log(1-q_h) + \log(q_h)\right)}{(1-\mu)\left(2H_{\frac{n}{2}} - 2H_n + \log(1-q_c) + \log(q_c)\right)} \tag{A3}$$

where H_m denotes the harmonic number of some m.
Notice that if (A2) holds, condition (A3) will hold, if:

$$1 > \frac{2H_{\frac{n}{2}} - 2H_n + \log(1-q_h) + \log(q_h)}{\left(2H_{\frac{n}{2}} - 2H_n + \log(1-q_c) + \log(q_c)\right)} \tag{A4}$$

or

$$\log(1-q_c) + \log(q_c) > \log(1-q_h) + \log(q_h) \tag{A5}$$

This is always the case, as $q_c < q_h$ if information avoidance exists.

Thus, with increasing n the range of payoffs for which information avoidance can arise becomes ceteris paribus larger.

Proof of increasing range for information avoidance with increasing n compared to Dictator treatment

Pivotality π_t for the dictator treatment is 1/3. Thus, if there are subjects avoiding information, it follows from (20) and with our parametrization, that for these subjects Δ_P lies within the range $[3\phi(\alpha_j), 6\psi(\beta_j)]$. The total length of this interval is thus $3(\phi - 2\psi)$.

The length of the corresponding interval for Voting11 and our parameterizations is $\frac{\phi}{252(1-q_c)^5 q_c^5} - \frac{\psi}{126(1-q_h)^5 q_h^5}$. Notice that $q_h < q_c$. Any possible interval would hence be smallest for the values $q_c = 0.5$ and $q_h \rightarrow q_c$. If we substitute these values, we get the minimum length of any interval for which under Voting11 information avoidance arises, which is $4(\phi - 2\psi) > 3(\phi - 2\psi)$. Thus, for any distribution of α and β in our sample, information avoidance will be more likely to arise in Voting11 than in the Dictator Treatment.

A.2 Experimental Instructions - On paper

To save space, we have merged the instructions into one text, highlighting the differences between the different treatment conditions in italics.

Dear participants,

Welcome to our experiment!

Please read the instructions carefully. The information given in the instructions is true. Your payment at the end of the experiment also depends on how well you have understood the instructions. The experiment as well as the analysis of the data are anonymous.
Please do not use any technical devices. If you have any questions during the experiment, please raise your hand – the experimenters will answer your question privately. Please do not talk to the other participants.
All expressions in the instructions refer equally to men and women.
This experiment consists of two parts. These instructions explain the first part of the experiment. Before the first part starts, each participant needs to answer some quiz questions on the instructions. As soon as all participants have finished the first part of the experiment, you will receive instructions for the second part.

Market

The first part of the experiment consists of a purchase decision which affects both your own payoff as well as a contribution to a carbon offset. The payment to the carbon offset will be made by the experimenters after the experiment is finished. You will receive more detailed information on carbon offsets on your screen at the beginning of the experiment.
You are endowed with 100 ECUs which you can spend on the virtual products A and B. In order to purchase, you need to click the button with the name of the product you want to purchase. You do not have the option not to buy.

Dictator

In the first part of the experiment, you will be split into groups of 3. You do not know the identity of your group members. It is your task to make an allocation decision. The allocation you have chosen determines your payoff, your group members' payoff as well as the contribution to a carbon offset. The payment to the carbon offset will be made by the experimenters after the experiment is finished. You will receive more detailed information on carbon offsets on your screen at the beginning of the experiment.

The allocation decision of one randomly determined group member will be implemented for the entire group. When you make your allocation decision, you do not know if you are the player whose allocation decision is implemented. This will be communicated as soon as all group members have made their allocation decisions. You need to decide between Allocation A and Allocation B. In order to select an allocation, you need to click the red button with the name of the preferred allocation. You do not have the option not to decide. The payment to the carbon offset and the payoffs for all group members are identical for each allocation.

Voting 3 (11)

In the first part of the experiment, you will be split into groups of 3 (11). You do not know the identity of your group members. It is your task to vote on the implementation of an allocation decision. The chosen allocation determines your payoff, your group members' payoff as well as the contribution to a carbon offset. The payment to the carbon offset will be made by the experimenters after the experiment is finished. You will receive more detailed information on carbon offsets on your screen at the beginning of the experiment.

You need to vote for the implementation of Allocation A or Allocation B. In order to vote for an allocation, you need to click the red button with the name of the preferred allocation. You do not have the option not to decide. The allocation that has received the majority of votes will be implemented, i.e. at least 2 (6) of the 3 (11) votes. The payment to the carbon offset and the payoffs for all group members are identical for each allocation.

Market with Full Information

The products differ in terms of their payoffs to the group members as well as in terms of their contributions to the offset. Both prices and contributions will be displayed on your screen.

Your earnings from a purchase decision are computed as your initial endowment minus the price you paid. Your earnings will be multiplied by 0.1 in order to compute your earnings in Euros. Analogously, the same of ECUs invested in carbon offsets will be multiplied by 0.1 to compute the contribution in Euros.

Market with Hidden Information

The products differ in terms of their payoffs to the group members as well as in terms of their contributions to the offset. For Product A, you know both the payoff and the contribution to the carbon offset (15 ECUs), whereas you only know the payoff of Product B. The possible contribution to the carbon offset of Product B can either be 0 or 30 ECUs, yet you do not know which number applies. Each contribution is equally likely.

Using the button "Contribution = 0 or 30 ECUs?" you have the opportunity to find out the contribution to the carbon offset of Product B. However, you can also make an allocation decision without information yourselves about B's contribution. Whether you use the button does not affect the contribution. Clicking the button has a cost of 0.10 ECUs. You can use the button only once.

Your earnings from a purchase decision are computed as your initial endowment minus the price you paid minus the costs of clicking the button in case you decided to click. Your earnings will be multiplied by 0.1 in order to compute your earnings in Euros. Analogously, the same of ECUs invested in carbon offsets will be multiplied by 0.1 in order to compute the contribution in Euros.

Hidden Information

The allocations differ in terms of their payoffs to the group members as well as in terms of their contribution to the offset. For Allocation A, you know both the payoff and the contribution to the carbon offset (15 ECUs), whereas you only know the payoff of Allocation B. The possible contributions to the carbon offset of allocation B can either be 0 or 30 ECUs, yet you do not know which number applies. Each contribution is equally likely.

Using the button "Contribution = 0 or 30 ECUs?" you have the opportunity to find out the contribution to the carbon offset of Allocation B. However, you can also make an allocation decision without information yourselves about B's contribution. Whether you use the button does not affect the contribution. Clicking the button has a cost of 0.10 ECUs. You can use the button only once.

Dictator and Voting Full Information

The allocations differ in terms of their payoffs to the group members as well as in terms of their contribution to the offset. Both payoffs and contributions for both allocations will be displayed on your screen.

Dictator

If your allocation decision is the one that is selected, your payoff and the payoff of your group members equals the payoff of the allocation that you have chosen. Similarly, the aggregate contribution to the offset equals the contribution of the selected allocation which is implemented for all group members. With 3 group members, this is three times the individual contribution. If your decision is not selected, it influences neither the payoff of the group members nor your own payoff. In this case, your payoff and your contribution to the carbon offset are determined by the allocation decisions of another group member. Please note: All group members always earn the same amount and contribute the same amount to the carbon offset. If the allocation of a player is implemented who chose Allocation A, 15 ECUs will be contributed for each group member will be contributed to the offset, i.e. in total 45.

Voting 3 (11)

Your payoff equals the payoff of the allocation for which the majority of the group members has voted. This is not the allocation for which you have voted if the majority of your group members has chosen differently. Analogously, your contribution to the carbon offset equals the contribution of the allocation which has received the majority of votes. Please note: All group members always earn the same amount and contribute the same amount to the carbon offset. If the allocation of a player is implemented who chose Allocation A, 15 ECUs will be contributed for each group member will be contributed to the offset.

Hidden Information

Your payoff will be determined by the implemented allocation minus the costs related to clicking the button if you decided to click. Your earnings will be multiplied by 0.1 in order to compute your earnings in Euros. Analogously, the same of ECUs invested in carbon offsets will be multiplied by 0.1 in order to compute the contribution in Euros.

The carbon offset has been selected by experts for climate policy at the University of Innsbruck and is certified according to the highest standards on the market. Certificates about the purchase of carbon offsets are available at the Institute of Public Finance at the University of Innsbruck.

At the end of the experiment, you will receive your earnings from part 1 and part 2 privately and in cash. In order to keep the process of payment as easy and time-efficient as possible, we will round your earnings towards the next higher 10 Cents.
As soon as all participants have completed the first part, you will receive new instructions on the second part.

Instructions on Part 2

Market

The second part of the experiment consists of 23 purchase decisions similar to the one you made in part 1. In each round, products differ in terms of their prices and in terms of their contributions to carbon offsets.

Market Full Information

Both the payoffs and the contributions for both products will be displayed on your screens.

Market Hidden Information

For Product A, you always know both the price and the contribution to the carbon offset, whereas you only know the price of Product B. The contributions of Product B can either be 0 or 30 ECUs, yet you do not know which number applies. Both contributions are equally likely. You have the option to reveal the contribution by clicking the button "Contribution = 0 or 30 ECUs?". Clicking the button costs 0.10 ECUs.

Dictator and Voting

The second part of the experiment consists of 23 allocation decisions similar to the one you made in part 1 of the experiment. In each round, new groups of 3 will be formed. You do not know the identity of your group members. After each round, new groups will be formed- Hence, you do not know who you are grouped with nor do you know if you have already interacted with your group members before.

Dictator

In each round, the allocation decision of one randomly selected group member will be implemented. When making the decision, you do not know if you will be the group member whose decision is implemented. This will only be communicated at the end of a round.

Voting 3 (11)

In each round, you need to vote for the implementation of Allocation A or Allocation B. In order to vote for one of the two allocations, you need to click the red button with the name of the allocation. You do not have the option not to vote. The allocation that has received the majority of vote, i.e. at least 2 (6) of 3 (11), will be implemented.

Hidden Information

Again allocations differ in the payoffs and in their contributions to carbon offsets. You know both the payoff and the contribution for Allocation A, while you only the payoff of Allocation B. The contributions of Allocation B can either be 0 or 30 ECUs, yet you do not know which number applies. With the button "Contribution = 0 or 30 ECUs?" you have the option to find out about the actual contribution of Allocation B. Clicking the button costs 0.10 ECUs.

Full Information

Again allocations differ in the payoffs and in their contributions to carbon offsets. Both the payoffs and the contributions for both options will be displayed on your screens.

Only one of the 23 decisions is payoff-relevant. The payoff-relevant round will be determined randomly at the end of the experiment. Again, ECUs will be multiplied by 0.1 to compute your earnings as well as the contribution to the carbon offset in Euros.
Your earnings from the experiment consist of your earnings from the first part and your earnings from the payoff-relevant round of part 2. Analogously, both contributions will be added to compute your contribution to the carbon offset.
This experiment will be terminated with a questionnaire. You will be informed about your earnings. Afterwards you will receive your earnings, rounded up to the next 10 Cents, privately and in cash.

A.3 Information on Offsets - On screen

Our consumption decisions are directly and indirectly associated with the emission of greenhouse gases. Greenhouse gases contribute to the global climate change. Among all greenhouse gases the impact of CO_2 on manmade climate change is largest.

There are suppliers which offer each individual the possibility to compensate their CO_2 emissions by making a compensation payment (=offset). The idea is to avoid CO_2 emissions elsewhere with the invested money. The money is invested in climate protection projects which e.g. plant trees or promote the usage of renewable energies.

Within this experiment, you will make allocation decisions in your group (**Market:** purchase decisions) which influence the amount of offsets purchased for this experiment. Hence, your decisions in the experiment will have actual consequences on the global amount of CO_2 emissions.

The offset-project has been selected by experts for climate policy at the University of Innsbruck. It is certified by the highest standards in the market.

For a price of €22 a ton of CO_2 emissions can be offset. The actual amount of CO_2 compensation depends on your choices.

A.4 Supplementary Analyses

A.4.1 Pivotality

Table A.1: Empirical frequency of voters being pivotal

	**Full Info**	**Hidden Info**
**Share of split votes**		
Voting3	0.378	0.457
Voting11	0.125	0.104
**No of rounds where a voter was pivotal**		
Voting3	9.07	10.97
Voting11	3	2.50
**Effect of a vote**		
Voting3	17.253	20.868
Voting11	20.625	17.188

With a p-value below 0.001 in χ^2-tests, the share of split votes (2 vs. 1) in Voting3 is significantly lower than the share of split votes (6 vs. 5) in Voting11 (see Table A.1). Accordingly, the average number of rounds in which a voter is pivotal in Voting3 exceeds the average number of rounds in Voting11. The average effect of a vote, i.e. the share of pivotal votes multiplied by the number of group members times the amount spent on offsets per person, varies between 17.188 and 20.868 across treatments, and is hence quite similar for all group treatments.

A.4.2 Power Analysis

For the power analysis, we consider χ^2-tests that compare two independent proportions in a cluster randomized design. In our design, each subject constitutes a cluster and the size of a cluster refers to the number of relevant decisions taken by each subject. For each treatment, we have 48 (44 in Voting11) participants, i.e. the number of clusters in each treatment is 48 or 44, respectively. Each subject played 24 rounds, of which 12 were characterized by a conflict of interests between the monetary and the green preferences of a subject. In half of these twelve rounds, the difference in payoffs was low, i.e. 5 or 10 ECUs. This yields six observations within each cluster for the comparisons of choices under low payoff differences. With a significance level of 5%, an intraclass correlation of 0.27, an effect size of 0.2 (0.6 vs. 0.4) and 44 clusters per treatment, the estimated statistical power to detect the exploitation of moral wiggle room is 0.916. For the detection of differences in the share of selfish choices between the difference institutional settings, the effect size is slightly smaller. Hence, the power lies slightly below 0.8 with an estimate of 0.797. Note that we have used conservative numbers for the power calculation.

A.4.3 Descriptive Statistics

Table A.2: Descriptive Statistics

	count	mean	sd	min	max
Male	376	0.44	0.50	0	1
Age	376	22.4	3.15	18	44
Econ	376	0.40	0.49	0	1
LeftRight	376	4.19	1.78	1	10
Honesty	376	3.36	1.59	1	9.40
ClimateIndividual	376	3.23	0.77	0.67	4.33
ClimateChangeFear	376	4.17	0.64	1	4.67
OffsetUse	376	1.65	0.84	1	4
OffsetNeg	376	3.05	1.18	1	5

Notes: Subjects self-classified as Male, Female, Diverse. Male is a dummy = 1 if the subject explicitly identifies as male. Econ is a dummy = 1 if the subject studies economics or business. LeftRight indicates the self-stated position on the political spectrum from 1 to 10, higher values = more right-wing orientation. Honesty captures self-stated honesty, higher value = more honest. Higher values of the variable ClimateIndividual indicate that the subject agrees with the statement that individual actions matter for climate change mitigation. Higher values of the variable ClimateChangeFear indicate that the subject is afraid of climate change. OffsetUse indicates the subject's experience with offsets (from 1 = never used to 4 = frequently used) and OffsetNeg captures if the subject holds a negative view towards offsets, higher values = more negative view.

A.4.4 Non-Parametric Test Results

Table A.3: p-values from clustered χ^2-tests: Share of Selfish Choices

**Full Information**	**Low Payoff Differences**	**High Payoff Differences**
I vs. V3	0.015	0.653
I vs. D	0.152	0.568
I vs. V11	0.702	0.613
D vs. V3	0.304	0.950
D vs. V11	0.288	0.311
V3 vs. V11	0.037	0.395
**Hidden Information**		
I vs. V3	0.535	0.663
I vs. D	0.811	0.649
I vs. V11	0.652	0.205
D vs. V3	0.721	0.970
D vs. V11	0.856	0.495
V3 vs. V11	0.848	0.448

Notes: p-values from clustered χ^2-tests (Donner, 1989) to take within-subject correlation into account.

Table A.4: p-values from clustered χ^2-tests: Moral Wiggle Room

**Full Information**	**Low Payoff Differences**	**High Payoff Differences**
Ind. Choice	0.001	0.478
Voting3	0.223	0.183
Dictator	0.058	0.133
Voting11	0.001	0.167

Notes: p-values from clustered χ^2-tests (Donner, 1989) to take within-subject correlation into account. The results are robust to controlling for multiple hypotheses testing using the Bonferroni-Holm method. Note that according to the non-parametric tests, also the difference between the share of selfish choices under full and hidden information in the Dictator treatment is significant at the 10% level. This is due to the lack of control variables: When performing the regression analysis for the Dictator condition without any controls, we also observe a significant effect (p = 0.095) of the 'Hidden' dummy, which disappears when we add controls.

A.4.5 Revelation Decisions

Figure 1 displays the average share of revelations for each institutional environment with hidden information, for aggregate data and separately for smaller and larger payoff differences. From the figure, it is obvious that the revelation rate is highest in the market setting and lowest for voting decisions in large groups — a finding that holds for all payoff differences. To test whether the observed results are statistically significant, we again perform several random effects panel regressions comparing the treatments in a pairwise manner (see Table A.5).[1] In (almost) all institutional settings, we find that in the later rounds, subjects become slightly more reluctant to reveal. Moreover, they reveal less often when the difference in payoffs is large. Considering the pairwise comparisons with the market setting as baseline, we only observe significantly fewer revelations in the Voting11 treatment; both the Dictator and the Voting3 treatment are statistically indistinguishable from the Market baseline and from each other, as Regression 4 shows. Interestingly, the higher revelation rate in the Market treatment does not manifest itself in a higher share of environmentally friendly choices as we find strong support for moral wiggle room exploitation in markets. Hence, in this treatment condition also curious egoists reveal information on the externalities, but do not incorporate it in their decisions in case they have discovered a conflict in interests. With significantly fewer revelations than in the Dictator treatment and in Voting3, Voting11 stands out, as it differs systematically from all other treatment conditions. This confirms our conjecture that information avoidance replaces expressive voting as a strategy to resolve cognitive dissonance in this case.

Figure 1: Share of revelations across treatments separated by price differences

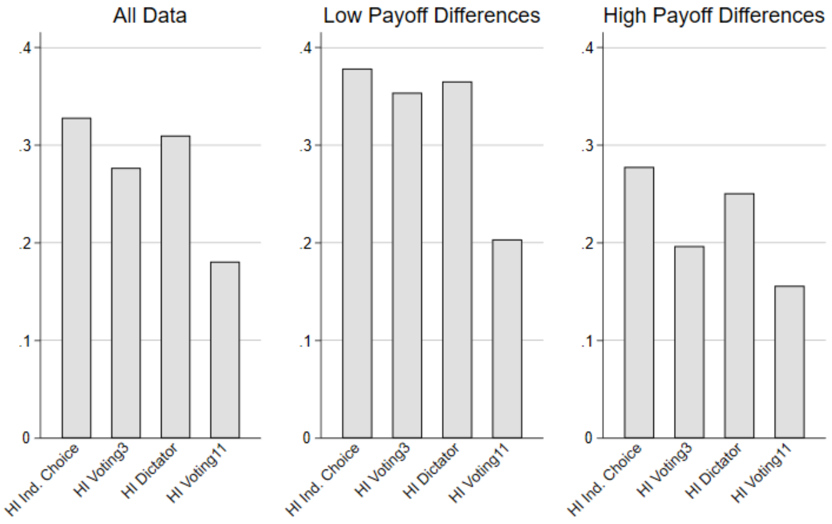

[1]For a regression analysis including a larger set of control variable see Table A.10 in section A.4.7 in the Appendix.

Table A.5: Revelation decisions

	(1) I vs. D	(2) I vs. V3	(3) I vs. V11	(4) D vs. V3	(5) D vs. V11	(6) V3 vs. V11
Period	-0.002	-0.000	-0.003*	-0.003*	-0.006***	-0.003**
	(0.002)	(0.002)	(0.002)	(0.002)	(0.001)	(0.001)
HighPD	-0.105***	-0.106***	-0.105***	-0.116***	-0.117***	-0.153***
	(0.030)	(0.030)	(0.030)	(0.029)	(0.029)	(0.030)
Dictator	0.018					
	(0.069)					
Dictator*HighPD	-0.011					
	(0.041)					
Voting3		0.010		-0.012		
		(0.073)		(0.075)		
Voting3*HighPD		-0.048		-0.037		
		(0.042)		(0.041)		
Voting11			-0.147**		-0.173**	-0.160**
			(0.062)		(0.071)	(0.065)
Voting11*HighPD			0.059		0.070*	0.107***
			(0.039)		(0.038)	(0.039)
Age	0.001	0.009	0.010	-0.003	-0.002	0.007
	(0.009)	(0.011)	(0.009)	(0.012)	(0.011)	(0.013)
Male	-0.074	-0.041	-0.019	-0.038	-0.026	0.012
	(0.069)	(0.070)	(0.070)	(0.065)	(0.066)	(0.065)
Econ	-0.071	-0.090	-0.124**	-0.074	-0.107	-0.118**
	(0.066)	(0.063)	(0.060)	(0.066)	(0.065)	(0.058)
Constant	0.437**	0.223	0.220	0.523*	0.547**	0.309
	(0.201)	(0.236)	(0.208)	(0.288)	(0.272)	(0.282)
R^2	0.028	0.038	0.063	0.036	0.058	0.058
N	2304	2304	2208	2304	2208	2208

Notes: Output from random-effects panel regressions. Standard errors (in parentheses) are clustered on subject-level. Dependent variable is a dummy which takes the value of 1 if information is revealed. Male is a dummy variable which takes the value of 1 if the subjects identified as male. Econ is a dummy variable that takes the values of 1 if the subject studies economics or business.

* $p < 0.10$, ** $p < 0.05$, *** $p < 0.01$

A.4.6 Supplementary Regressions

Table A.6: Selfish choices under full information in comparison to Market and to Voting11

	(1) M vs. other treatments	(2) V11 vs. other treatments
Period	0.017***	0.017***
	(0.005)	(0.005)
Period2	-0.000**	-0.000**
	(0.000)	(0.000)
HighPD	0.354***	0.322***
	(0.044)	(0.041)
Ind. Choice		-0.022
		(0.072)
Ind. Choice*HighPD		0.032
		(0.060)
Voting3	0.180**	0.158**
	(0.076)	(0.072)
Voting3*HighPD	-0.203***	-0.170***
	(0.060)	(0.057)
Dictator	0.140*	0.118*
	(0.074)	(0.072)
Dictator*HighPD	-0.171***	-0.138**
	(0.059)	(0.056)
Voting11	0.022	
	(0.072)	
Voting11*HighPD	-0.032	
	(0.060)	
Age	0.008	0.008
	(0.008)	(0.008)
Male	0.133***	0.133***
	(0.046)	(0.046)
Econ	0.025	0.025
	(0.049)	(0.049)
Constant	0.008	0.030
	(0.177)	(0.181)
R^2	0.117	0.117
N	2256	2256

Notes: Output from random-effects panel regression. Standard errors (in parentheses) clustered on subject-level. Dependent variable is a dummy = 1 if the selfish option is chosen. Male is a dummy = 1 if the subject identified as male. Econ is a dummy = 1 if the subject studies economics or business. * $p < 0.10$, ** $p < 0.05$, *** $p < 0.01$

A.4.7 Regressions Including a Larger Set of Control Variables

Table A.7: Selfish Choices under Full Information

	(1) I vs. D	(2) I vs. V3	(3) I vs. V11	(4) D vs. V3	(5) D vs. V11	(6) V3 vs. V11
Period	0.002	0.004**	0.010***	0.005**	0.011***	0.013***
	(0.002)	(0.002)	(0.002)	(0.002)	(0.003)	(0.002)
Dictator	0.142**					
	(0.072)					
HighPD	0.372***	0.365***	0.345***	0.195***	0.176***	0.144***
	(0.046)	(0.045)	(0.045)	(0.039)	(0.039)	(0.039)
Dictator*HighPD	-0.171***					
	(0.059)					
Voting3		0.153**		0.027		
		(0.077)		(0.078)		
Voting3*HighPD		-0.207***		-0.037		
		(0.061)		(0.057)		
Voting11			0.008		-0.103	-0.154**
			(0.068)		(0.073)	(0.073)
Voting11*HighPD			-0.021		0.142**	0.172***
			(0.060)		(0.056)	(0.056)
Age	-0.012	0.022*	-0.004	0.029***	0.006	0.023*
	(0.011)	(0.013)	(0.006)	(0.011)	(0.007)	(0.012)
Male	0.080	0.041	0.055	0.110	0.168**	0.108
	(0.069)	(0.062)	(0.060)	(0.073)	(0.078)	(0.066)
Econ	-0.004	0.042	-0.053	-0.011	-0.134**	-0.051
	(0.072)	(0.064)	(0.057)	(0.073)	(0.067)	(0.062)
LeftRight	0.006	0.016	0.005	0.008	0.004	0.013
	(0.019)	(0.017)	(0.016)	(0.017)	(0.016)	(0.016)
GenHonesty	-0.001	0.035*	0.037**	0.009	0.014	0.048**
	(0.020)	(0.019)	(0.015)	(0.020)	(0.018)	(0.019)
ClimateIndividual	-0.143***	-0.075*	-0.040	-0.097**	-0.075**	-0.035
	(0.034)	(0.043)	(0.040)	(0.045)	(0.038)	(0.042)
ClimateChangeFear	-0.000	0.043	-0.025	0.014	-0.038	-0.038
	(0.042)	(0.058)	(0.044)	(0.044)	(0.039)	(0.046)
OffsetUse	0.009	-0.064*	-0.047	-0.031	-0.007	-0.070*
	(0.036)	(0.036)	(0.031)	(0.044)	(0.041)	(0.040)
OffsetNeg	0.010	0.020	0.038*	-0.033	-0.015	-0.011
	(0.023)	(0.026)	(0.022)	(0.027)	(0.026)	(0.027)
Constant	0.978**	-0.264	0.396	0.117	0.613**	0.067
	(0.381)	(0.456)	(0.243)	(0.416)	(0.282)	(0.403)
R^2	0.148	0.164	0.200	0.141	0.174	0.208
N	1152	1152	1104	1152	1104	1104

Notes: Output from random-effects panel regressions. Standard errors (in parentheses) clustered on subject-level. Dependent variable is a dummy = 1 if the selfish option is chosen. Male is a dummy = 1 if the subject identified as male. Econ is a dummy = 1 if the subject studies economics or business. LeftRight indicates the self-stated position on the political spectrum from 1 to 10, higher values = more right-wing orientation. Honesty captures self-stated honesty, higher value = more honest. Higher values of the variable ClimateIndividual indicate that the subject agrees with the statement that individual actions matter for climate change mitigation. Higher values of the variable ClimateChangeFear indicate that the subject is afraid of climate change. OffsetUse indicates the subject's experience with offsets (from 1 = never used to 4 = frequently used) and OffsetNeg captures if the subject holds a negative view towards offsets, higher values = more negative view. * $p < 0.10$, ** $p < 0.05$, *** $p < 0.01$

Table A.8: Selfish Choices under Hidden Information

	(1) I vs. D	(2) I vs. V3	(3) I vs. V11	(4) D vs. V3	(5) D vs. V11	(6) V3 vs. V11
Period	0.003	0.002	0.007***	0.003*	0.009***	0.008***
	(0.002)	(0.002)	(0.002)	(0.002)	(0.002)	(0.002)
Dictator	-0.057					
	(0.070)					
HighPD	0.139***	0.141***	0.125***	0.194***	0.186***	0.139***
	(0.034)	(0.034)	(0.034)	(0.035)	(0.035)	(0.043)
Dictator*HighPD	0.055					
	(0.049)					
Voting3		-0.041		0.071		
		(0.069)		(0.077)		
Voting3*HighPD		0.009		-0.045		
		(0.055)		(0.056)		
Voting11			-0.004		0.102	0.047
			(0.062)		(0.073)	(0.070)
Voting11*HighPD			0.041		-0.020	0.026
			(0.054)		(0.054)	(0.060)
Age	-0.000	-0.007	-0.004	0.007	0.009	0.002
	(0.008)	(0.008)	(0.008)	(0.011)	(0.010)	(0.013)
Male	0.040	0.159**	0.090	0.122*	0.035	0.114*
	(0.067)	(0.064)	(0.060)	(0.063)	(0.056)	(0.059)
Econ	0.098	0.141**	0.101*	0.019	0.031	0.047
	(0.065)	(0.062)	(0.055)	(0.072)	(0.066)	(0.056)
LeftRight	-0.011	-0.003	-0.009	0.009	0.013	0.011
	(0.015)	(0.014)	(0.015)	(0.015)	(0.015)	(0.013)
GenHonesty	0.026	0.024	0.010	0.008	0.009	-0.004
	(0.016)	(0.020)	(0.017)	(0.020)	(0.015)	(0.018)
ClimateIndividual	-0.051	0.039	-0.013	-0.034	-0.069*	-0.024
	(0.044)	(0.045)	(0.047)	(0.040)	(0.040)	(0.039)
ClimateChangeFear	-0.034	-0.022	0.010	-0.122**	-0.079	-0.040
	(0.049)	(0.047)	(0.040)	(0.053)	(0.052)	(0.055)
OffsetUse	-0.040	-0.045	-0.000	-0.044	-0.014	-0.009
	(0.050)	(0.030)	(0.033)	(0.037)	(0.037)	(0.025)
OffsetNeg	0.015	-0.037*	-0.023	0.029	0.028	-0.007
	(0.030)	(0.021)	(0.024)	(0.030)	(0.033)	(0.024)
Constant	0.836**	0.757**	0.661**	0.842**	0.630*	0.665**
	(0.334)	(0.314)	(0.293)	(0.364)	(0.329)	(0.297)
R^2	0.078	0.092	0.085	0.112	0.127	0.095
N	1152	1152	1104	1152	1104	1104

Notes: Output from random-effects panel regressions. Standard errors (in parentheses) clustered on subject-level. Dependent variable is a dummy = 1 if the selfish option is chosen. Male is a dummy = 1 if the subject identified as male. Econ is a dummy = 1 if the subject studies economics or business. LeftRight indicates the self-stated position on the political spectrum from 1 to 10, higher values = more right-wing orientation. Honesty captures self-stated honesty, higher value = more honest. Higher values of the variable ClimateIndividual indicate that the subject agrees with the statement that individual actions matter for climate change mitigation. Higher values of the variable ClimateChangeFear indicate that the subject is afraid of climate change. OffsetUse indicates the subject's experience with offsets (from 1 = never used to 4 = frequently used) and OffsetNeg captures if the subject holds a negative view towards offsets, higher values = more negative view. * $p < 0.10$, ** $p < 0.05$, *** $p < 0.01$

Table A.9: Moral Wiggle Room Exploitation

	(1) IL	(2) V3L	(3) DL	(4) V11L
Period	0.001	0.005**	0.003	0.018***
	(0.002)	(0.002)	(0.002)	(0.002)
HighPD	0.370***	0.158***	0.197***	0.318***
	(0.046)	(0.040)	(0.039)	(0.039)
Hidden	0.266***	0.092	0.065	0.266***
	(0.066)	(0.075)	(0.079)	(0.065)
HighPD*Hidden	-0.226***	-0.012	-0.004	-0.153***
	(0.055)	(0.059)	(0.053)	(0.056)
Age	-0.016*	0.028*	0.005	0.002
	(0.009)	(0.015)	(0.009)	(0.007)
Male	0.045	0.135**	0.078	0.095
	(0.057)	(0.065)	(0.070)	(0.070)
Econ	0.152**	0.107*	-0.033	-0.039
	(0.061)	(0.064)	(0.072)	(0.058)
LeftRight	-0.019	0.016	0.014	0.009
	(0.017)	(0.016)	(0.015)	(0.015)
GenHonesty	0.029*	0.035*	0.003	0.018
	(0.017)	(0.021)	(0.017)	(0.016)
ClimateIndividual	-0.028	0.013	-0.134***	-0.015
	(0.041)	(0.041)	(0.034)	(0.038)
ClimateChangeFear	0.024	-0.048	-0.046	-0.057
	(0.042)	(0.055)	(0.040)	(0.035)
OffsetUse	-0.061*	-0.066*	0.014	-0.012
	(0.034)	(0.034)	(0.051)	(0.030)
OffsetNeg	-0.002	-0.026	0.011	0.006
	(0.022)	(0.024)	(0.030)	(0.026)
Constant	0.722*	-0.092	0.833**	0.296
	(0.375)	(0.448)	(0.325)	(0.221)
R^2	0.145	0.149	0.147	0.214
N	1152	1152	1152	1056

Notes: Output from random-effects panel regressions. Standard errors (in parentheses) clustered on subject-level. Dependent variable is a dummy = 1 if the selfish option is chosen. Male is a dummy = 1 if the subject identified as male. Econ is a dummy = 1 if the subject studies economics or business. LeftRight indicates the self-stated position on the political spectrum from 1 to 10, higher values = more right-wing orientation. Honesty captures self-stated honesty, higher value = more honest. Higher values of the variable ClimateIndividual indicate that the subject agrees with the statement that individual actions matter for climate change mitigation. Higher values of the variable ClimateChangeFear indicate that the subject is afraid of climate change. OffsetUse indicates the subject's experience with offsets (from 1 = never used to 4 = frequently used) and OffsetNeg captures if the subject holds a negative view towards offsets, higher values = more negative view. * $p < 0.10$, ** $p < 0.05$, *** $p < 0.01$

Table A.10: Revelation Decisions

	(1) I vs. D	(2) I vs. V3	(3) I vs. V11	(4) D vs. V3	(5) D vs. V11	(6) V3 vs. V11
Period	-0.002	-0.000	-0.003*	-0.003*	-0.006***	-0.003**
	(0.002)	(0.002)	(0.002)	(0.002)	(0.001)	(0.001)
Dictator	0.014					
	(0.067)					
HighPD	-0.105***	-0.106***	-0.105***	-0.116***	-0.117***	-0.153***
	(0.030)	(0.030)	(0.030)	(0.029)	(0.029)	(0.030)
Dictator*HighPD	-0.011					
	(0.041)					
Voting3		0.014		-0.027		
		(0.069)		(0.073)		
Voting3*HighPD		-0.048		-0.038		
		(0.042)		(0.042)		
Voting11			-0.164***		-0.149**	-0.201***
			(0.059)		(0.074)	(0.065)
Voting11*HighPD			0.059		0.070*	0.107***
			(0.039)		(0.038)	(0.039)
Age	0.006	0.005	0.011	-0.003	0.003	0.003
	(0.008)	(0.010)	(0.009)	(0.012)	(0.011)	(0.014)
Male	-0.038	-0.048	0.004	0.016	0.022	0.059
	(0.067)	(0.065)	(0.072)	(0.069)	(0.068)	(0.063)
Econ	-0.077	-0.082	-0.105*	-0.040	-0.067	-0.070
	(0.067)	(0.069)	(0.057)	(0.075)	(0.066)	(0.058)
LeftRight	0.001	-0.012	-0.007	-0.036***	-0.032**	-0.044***
	(0.015)	(0.014)	(0.016)	(0.013)	(0.016)	(0.012)
GenHonesty	-0.057***	-0.024	-0.009	-0.037*	-0.027	0.012
	(0.017)	(0.021)	(0.017)	(0.021)	(0.018)	(0.017)
ClimateIndividual	0.005	0.009	-0.002	0.015	-0.007	0.035
	(0.045)	(0.049)	(0.058)	(0.041)	(0.048)	(0.044)
ClimateChangeFear	0.018	0.010	0.047	-0.032	0.029	-0.018
	(0.054)	(0.049)	(0.054)	(0.058)	(0.058)	(0.055)
OffsetUse	0.009	0.012	-0.031	0.023	-0.018	-0.011
	(0.053)	(0.036)	(0.034)	(0.042)	(0.040)	(0.025)
OffsetNeg	0.018	0.063**	0.027	-0.008	-0.034	0.003
	(0.033)	(0.026)	(0.030)	(0.032)	(0.033)	(0.026)
Constant	0.331	0.160	0.036	0.850**	0.652	0.473
	(0.374)	(0.368)	(0.334)	(0.430)	(0.402)	(0.342)
R^2	0.071	0.075	0.080	0.078	0.089	0.091
N	2304	2304	2208	2304	2208	2208

Notes: Output from random-effects panel regressions. Standard errors (in parentheses) clustered on subject-level. Dependent variable is a dummy = 1 if information is revealed. Male is a dummy = 1 if the subject identified as male. Econ is a dummy = 1 if the subject studies economics or business. LeftRight indicates the self-stated position on the political spectrum from 1 to 10, higher values = more right-wing orientation. Honesty captures self-stated honesty, higher value = more honest. Higher values of the variable ClimateIndividual indicate that the subject agrees with the statement that individual actions matter for climate change mitigation. Higher values of the variable ClimateChangeFear indicate that the subject is afraid of climate change. OffsetUse indicates the subject's experience with offsets (from 1 = never used to 4 = frequently used) and OffsetNeg captures if the subject holds a negative view towards offsets, higher values = more negative view. * $p < 0.10$, ** $p < 0.05$, *** $p < 0.01$

A.4.8 Figures

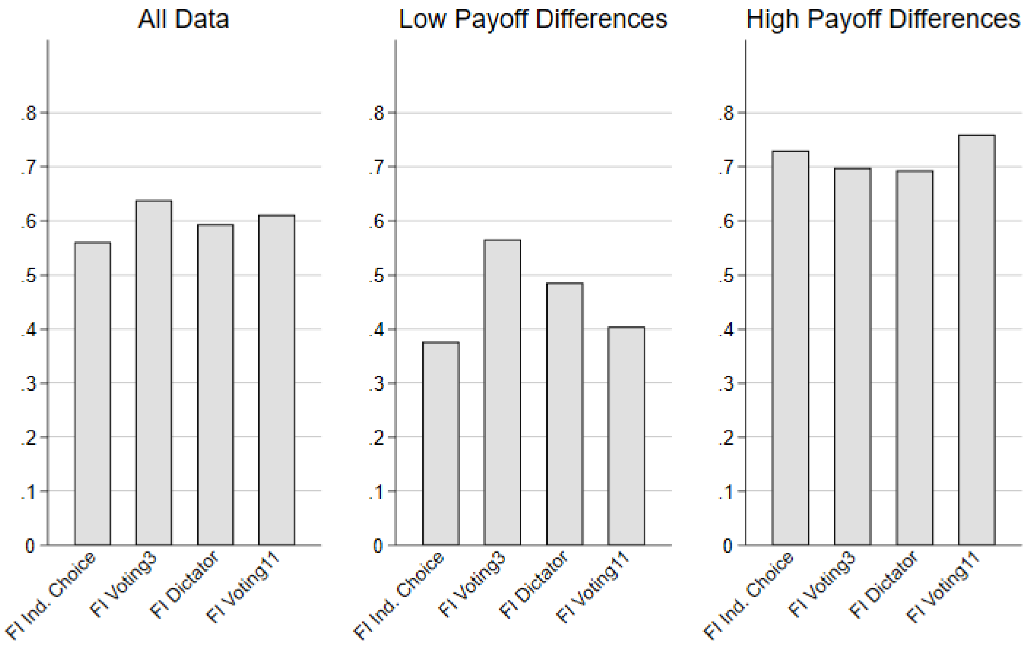

Figure 2: Share of selfish choices in conflict situations under full information

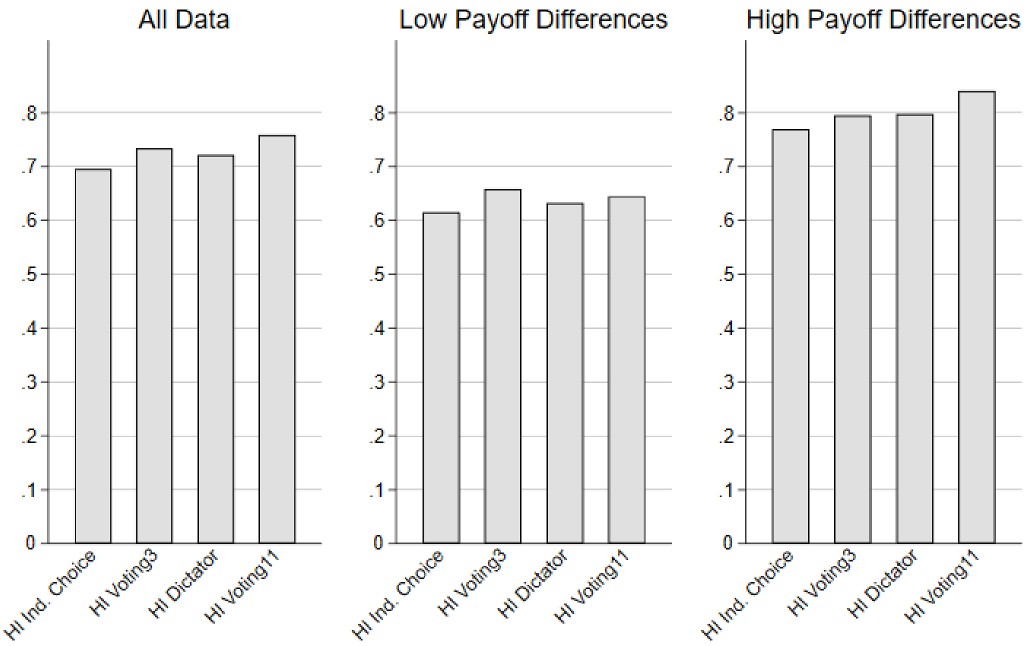

Figure 3: Share of selfish choices in conflict situations under hidden information

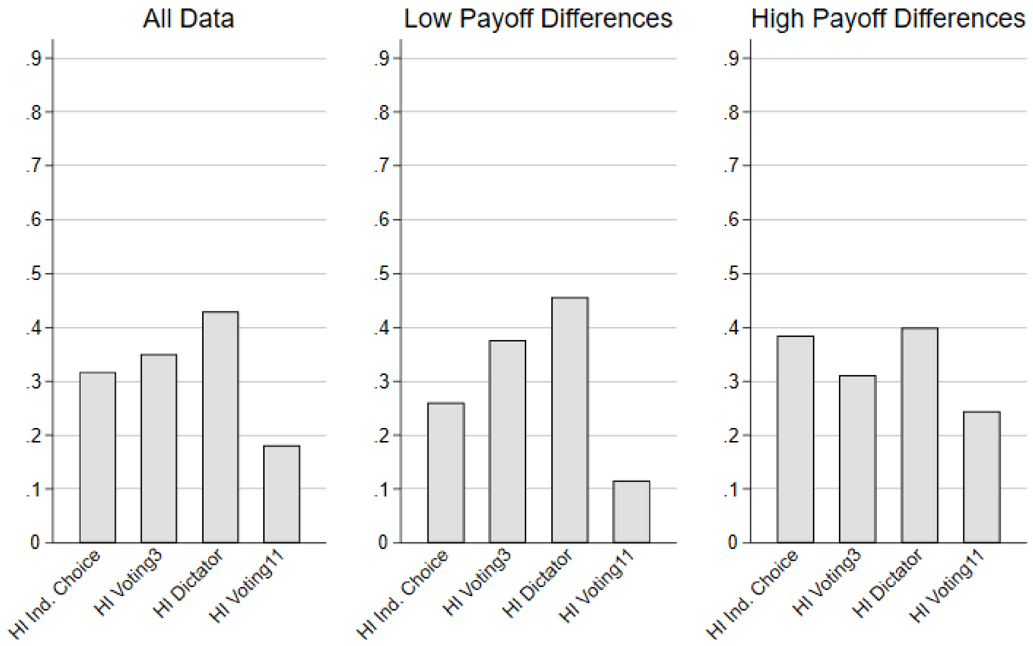

Figure 4: Share of selfish choices in conflict situations under hidden information – *informed* subjects

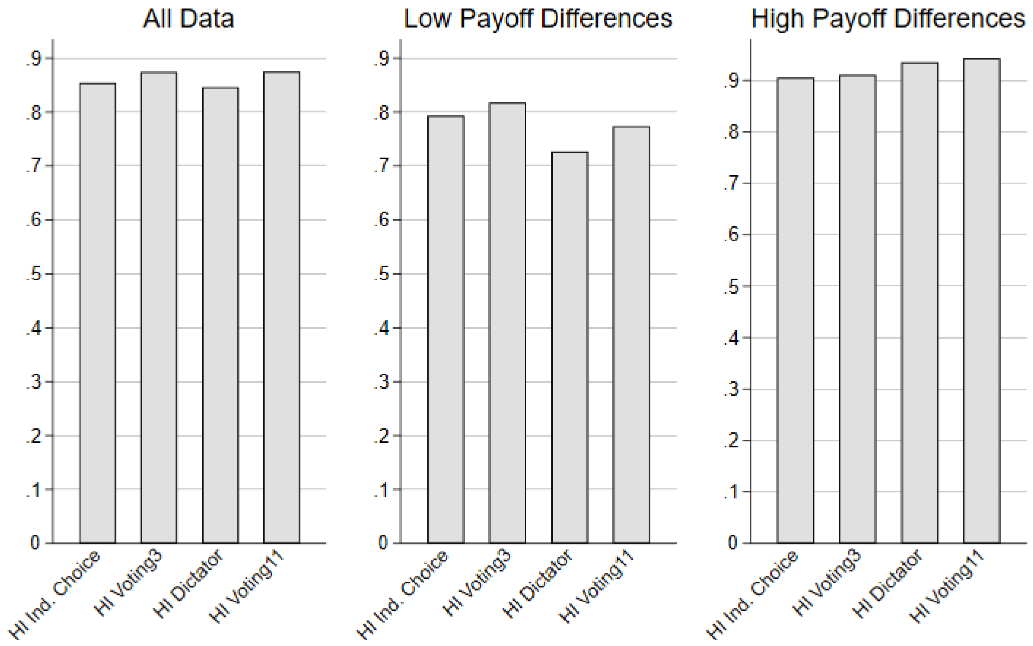

Figure 5: Share of selfish choices in conflict situations under hidden information – *uninformed* subjects
